# 3′-Caffeoylquercetin Glycosides and 4′-Caffeoylkaempferol Glycosides—Novel Antioxidant Flavonoids Discovered in the Freesia Yellow Flowers

**DOI:** 10.3390/antiox14020158

**Published:** 2025-01-28

**Authors:** Kazutoshi Shindo, Nozomi Iwamoto, Mayu Usami, Ayuna Saito, Miho Sato, Maho Sugaya, Nao Miyashita, Minoru Murahama, Yasuki Higashimura, Miho Takemura, Kazuo Furihata, Norihiko Misawa

**Affiliations:** 1 Department of Food and Nutrition, Japan Women’s University, 2-8-1 Mejirodai, Bunkyo-ku, Tokyo 112-8681, Japan; 2 Ishikawa Agriculture and Forestry Research Center, 295-1 Saida-machi, Kanazawa 920-3198, Japan; n-miya@pref.ishikawa.lg.jp (N.M.); murahama@pref.ishikawa.lg.jp (M.M.); 3 Department of Food Science, Ishikawa Prefectural University, 1-308 Suematsu, Nonoichi-shi 921-8836, Japan; yasuki@ishikawa-pu.ac.jp; 4 Research Institute for Bioresources and Biotechnology, Ishikawa Prefectural University, 1-308 Suematsu, Nonoichi-shi 921-8836, Japan; mtake@ishikawa-pu.ac.jp; 5 Division of Agriculture and Agricultural Life Sciences, The University of Tokyo, 1-1-1, Yayoi, Bunkyo-ku, Tokyo 113-8657, Japan; afuriha@g.ecc.u-tokyo.ac.jp

**Keywords:** freesia petals, quercetin glycosides, kaempferol glycosides, caffeic acid, edible flower, antioxidant activity

## Abstract

The petals of flowering plants should retain unique antioxidants that have not been found in the fruits, as the petals need to stay open to attract pollinators against photooxidation and devise a solution to avoid eating attacks. We reported that the yellow petals of freesia cultivars (*Freesia* x *hybrida*) accumulated original apocarotenoids, mono- and di-neapolitanosyl crocetin. Here, in the yellow petals, we discovered eight novel flavonoids by their structural determination, including four 3′-caffeoylquercetin 3,7-glycosides, one 3′-caffeoylquercetin 3-glycoside, and three 4′-caffeoylkaempferol 3,7-glycosides. The 3-carbon sugar part was a minor hexose dimer [D-glucosyl-D-glucoside or D-glucosyl-L-rhamnoside] with the β1,2-linkage, while the 7-carbon was usually O-glycosylated with D-glucose, L-rhamnose, or D-glucuronic acid. Such caffeoyl-flavonol glycosides were also present in freesia white petals, regardless of the cultivars and wild species. When dihydroflavonols, the last common precursors between flavonols and anthocyanins, switch to the flavonol route, these caffeoyl-flavonol glycosides are likely to be synthesized via quercetin or kaempferol. All the eight flavonoids exerted in vitro antioxidant activities against both lipid peroxidation and radical generation. Specifically, 3′-caffeoylquercetin 3-sophoroside and its 7-glucuronide showed superior antioxidant activity. Freesia yellow and white flowers have been utilized as edible flowers, indicating the importance of evaluating the human benefits and risks of newly identified flavonoids.

## 1. Introduction

Higher plants, especially ornamental flowering plants, have petals that biosynthesize diverse low-molecular-weight chemicals responsible for their wide variety of colors, including carotenoids, flavonoids (including anthocyanins), and betalains [1,2,3,4]. Petals also attract pollinators [5]. To increase the frequency of pollination, they need to stay open for a longer time against photooxidation and devise a solution to stop herbivores from eating them, e.g., they may accumulate repellents or chemicals poisonous to herbivores [6]. Therefore, petals are unlikely to be tasty or sweet, which differs from the gustatory characteristics of the fruits of higher plants. These considerations imply the existence of unique antioxidant chemicals in flowering plant petals which have not been found in fruits, for example, the diacylated delphinidin-based anthocyanin gentiodelphin existing in gentian flowers [2].

Limited numbers of flowering plants have traditionally been used as edible flowers. For example, Matyjaszczyk and Śmiechowska (2019) introduced 38 plant flowers, including black elder, pansy, cornflower, chrysanthemum, sweet violet, nasturtium, rose, and freesia, as the flowers most often used as food and their applications [6]. It has also been demonstrated that low-molecular-weight antioxidative compounds, mainly pigments, of edible flower origin can be bioactive compounds affecting the health of animals, including humans [4,6,7,8].

Freesia (the Iridaceae family) is a flowering plant that constitutes several wild species (e.g., *Freesia refracta*, *Freesia leichtlinii*, and *Freesia caryophyllaceae*) of South African (the Cape Province area) origin and many cultivars (*Freesia* x *hybrida*) that have been generated by crossing [9,10]. Freesia cultivars are mainly marketed not as edible flowers but as ornamental cut flowers. The cultivars exhibit diverse flower colors, i.e., red, orange, yellow, white, pink, blue, and purple. A considerable portion of these color patterns, including blue and purple, is attributed to the formation of anthocyanins, or proanthocyanidins (partially exist) [11]. On the other hand, cultivars with yellow petals are the most cultivated plants among the freesia cultivars, e.g., they maintain an 80% share of the market in Japan. Recently, we clarified that the yellow pigments in the yellow petals of freesia (cultivars ‘f2’, ‘Aladin’, ‘Kayak’, ‘Passat’, and ‘Boulevard’) are original water-soluble apocarotenoids, crocetin (mono)neapolitanosyl ester and crocetin dineapolitanosyl ester [12].

We noticed the existence of other water-soluble colorless compounds with antioxidant activity in yellow freesia flowers during preparation experiments of the apocarotenoids. The main purpose of the present study is the identification of the water-soluble colorless antioxidants. Here, we showed that these antioxidants are caffeoyl-flavonol glycosides, which are produced in the yellow petals of the freesia cultivars and also in the white petals of freesia (cultivars ‘f10’ and ‘f11’) and wild species (*F. refracta* and *F. leichtlinii* ‘Alba’). These flavonoid glycosides were purified from methanol and 50% methanol extracts of yellow flowers, and their chemical structures were determined to be novel caffeoyl-flavonol glycosides. We also implied their biosynthetic pathway and further measured their in vitro antioxidant activity.

## 2. Materials and Methods

### 2.1. Plant Materials

**Figure 1:**
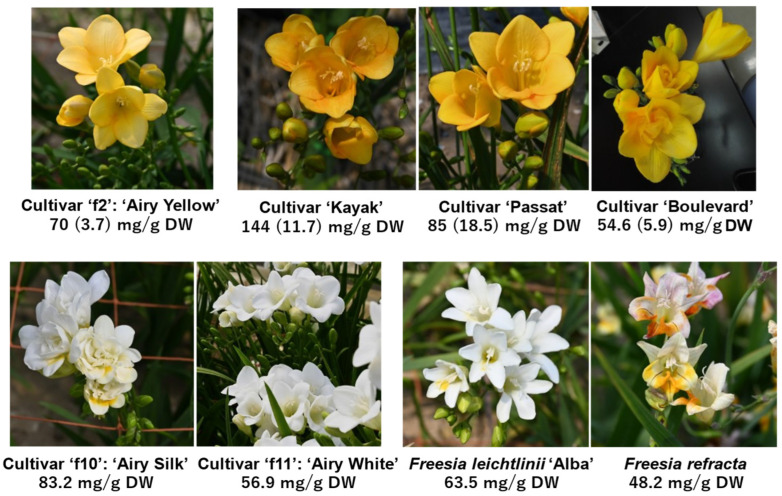
Freesia plants used in this study. We selected six *Freesia* x *hybrida* cultivars and two wild species *Freesia leichtlinii* ‘Alba’ and *Freesia refracta*. Amounts of total flavonoids, and total apocarotenoids (parenthesized) only in yellow petals, are shown under the photographs.

The freesia plants with yellow petals used in this study were *Freesia* x *hybrida* cultivars ‘Ishikawa f2 go’ (‘f2’), ‘Kayak’, ‘Passat’, and ‘Boulevard’ (Figure 1). Cultivar ‘f2’, named ‘Airy Yellow’, was bred in Ishikawa Prefecture by crossing cultivars ‘Aladin’ and ‘Rapid Yellow’ [13]. We further used cultivars ‘Ishikawa f10 go’ (‘f10’), named ‘Airy Silk’, and ‘Ishikawa f11 go’ (‘f11’), named ‘Airy White’, with white petals (Figure 1), which were recently developed in Ishikawa Prefecture. Wild species *Freesia leichtlinii* subsp. alba (also classified as *Freesia alba*; here called ‘Alba’) and *F. refracta* (Figure 1) were also used in this study. These plants were grown in the greenhouses of the Ishikawa Agriculture and Forestry Research Center and Ishikawa Prefectural University from October, and their flowers were harvested between mid-March and early April and stored in a freezer until use.

### 2.2. Solvents and Reagents

Analytical grade dichloromethane (CH_2_Cl_2_), *n*-hexane, ethanol (EtOH), methanol (MeOH), acetonitrile (CH_3_CN), ethyl acetate (EtOAc), and acetate (AcOH) were purchased from Kanto Chemical Co., Inc. (Tokyo, Japan). Frozen brains of 8-week-old male Wistar rats were purchased from Funakoshi Co., Ltd. (Tokyo, Japan). All other reagents were purchased from Sigma-Aldrich (St. Louis, MO, USA).

### 2.3. Spectroscopic Analysis Using Nuclear Magnetic Resonance (NMR) and Mass Spectrometry (MS)

NMR spectra [^1^H and ^13^C NMR, Double Quantum Filtered Correlation Spectroscopy (DQF COSY), Heteronuclear Single Quantum Correlation (HSQC), J-resolved HSQC, Heteronuclear Multiple Bond Coherence (HMBC), and Nuclear Overhauser Effect Spectroscopy (NOESY)] were obtained using a Bruker AVANCE400 Spectrometer and standard programs in TopSpin1.3 software. The chemical shifts were referenced to the solvent signals (DMSO-*d*_6_: δ_H_ = 2.49, δ_C_ = 39.7). High-resolution electrospray ionization MS (HR-ESI-MS) spectra were obtained using a JMS-T100LP instrument (JEOL, Tokyo, Japan), and the accumulated mass was calibrated using reserpine (C_33_H_39_N_2_O_9_: *m*/*z* 607.2655544 (M − H)^−^ or C_33_H_41_N_2_O_9_: *m*/*z* 609.2812044 (M + H)^+^).

### 2.4. Isolation of Flavonoid Compounds from the ‘f2’ Flowers

Copious amounts of the harvested ‘f2’ flowers were dried for two months indoors at room temperature, sometimes with the use of a dehumidification machine (IRIS Ohyama efeel; compressor type) to generate dry flowers with approximately 1/10 weight compared with that of the raw flowers. Dried freesia flowers (18.6 g) were powdered using a mill, extracted by adding 1 L of CH_2_Cl_2_-MeOH (1:1) with stirring for 30 min at room temperature under dim light, and then filtered under reduced pressure. MeOH (1 L) was then added to the filtered debris and extracted in the same manner. Subsequently, 1 L of 50% (*v*/*v*) MeOH (1 L) was added to the debris and treated in the same manner.

The MeOH and 50% MeOH extracts were mixed and concentrated to dryness to obtain a pale yellow oil (3.95 g). This oil was further purified using preparative octadecylsilyl silica gel (ODS) high-performance liquid chromatography (HPLC). The HPLC conditions were as follows: column, ADME-HR S5 (Osaka Soda Co., Ltd., Osaka, Japan) of i.d. 10 × 250 mm; solvent, 20% CH_3_CN (*v*/*v*) containing 0.1% trifluoroacetic acid (*v*/*v*); flow rate, 3.0 mL/min; and detector, photodiode array (PDA) (200–500 nm). In this chromatography, compounds **1**–**6** were eluted as pure compounds (**1**: *t_R_* 9.2 min (33.0 mg), **2**: *t_R_* 9.8 min (20.2 mg), **3**: *t_R_* 12.6 min (21.7 mg), **4**: *t_R_* 19.3 min (76.1 mg), **5**: *t_R_* 22.5 min (61.0 mg), **6**: *t_R_* 30.0 min (41.5 mg)) (Figure S1).

### 2.5. Isolation of Flavonoid Compounds from the ‘Kayak’ Flowers

‘Kayak’ flowers (146.7 g) were freeze-dried and powdered using a mill (12.8 g). The powder was extracted using the method described above to obtain a pale yellow oil (5.91 g). This oil was further purified using preparative ODS HPLC. The HPLC conditions were as follows: column, Develosil C30-UG (Nomura Chemical. Co., Ltd., Aichi, Japan) with an i.d. of 20 × 250 mm; solvent, 17% CH_3_CN (*v*/*v*) containing 0.1% trifluoroacetic acid (*v*/*v*); flow rate, 8.0 mL/min; and detector, PDA (200–500 nm). In this chromatography, compounds **7** and **8** were eluted as pure compounds (**7**: *t_R_* 10.0 min (428.3 mg), **8**: *t_R_* 12.0 min (589.7 mg)) (Figure S2).

### 2.6. Analysis of Total Flavonoid Compounds in Individual Freesia Cultivars and Wild Species

We extracted eight frozen petals of freesia cultivars ‘f2’, ‘Kayak’, ‘Passat’, ‘Boulevard’, ‘f10’, ‘f11’, ‘Alba’, and *F. refracta* (each 30–50 g), with MeOH and 50% (*v*/*v*) MeOH using a similar method to that described above and analyzed them using ODS HPLC (HPLC conditions: column, pegasil ODS SP100 (Senshu Chemical. Co., Ltd., Tokyo, Japan) of i.d. 4.6 × 250 mm; solvent A: 5% (*v*/*v*) CH_3_CN + 20 mM phosphoric acid, B: 95% (*v*/*v*) CH_3_CN + 20 mM phosphoric acid. 0 → 3 min A 100%, 3 → 20 min A 100% → B 100% linear gradient, 20 → 30 min B 100%; flow rate, 1.0 mL/min; detector, PDA (200–500 nm and 330 nm)). The total amount of flavonoid glycosides in each freesia species was calculated by integrating the areas of the individual flavonoid glycoside peaks at 330 nm.

### 2.7. Purification of Glucose from Compound ***5*** Using Acid Hydrolysis

Compound **5** (15.0 mg) was suspended in 5.0 mL of 2.0 N HCl and heated in reflux for 2 h. After removing the solvent in vacuo, the reaction mixture was subjected to silica gel column chromatography (i.d. 10 × 150 mm) (silica 60, Kanto Chemical. Co., Inc., Tokyo, Japan) in CH_2_Cl_2_: MeOH (2:1) to obtain pure glucose (2.2 mg).

### 2.8. Determination of L-Rhamnose in Compound ***4*** by L-Rhamnosidase

Compound **4** (1.0 mg) was dissolved in 0.1 M phosphate buffer (pH 7.5) (1 mL) and allowed to react with 1 mL of α L-rhamnosidase from *Thermomicrobacterium* strain PRI-1686 (Funakoshi Co., Ltd., Tokyo, Japan) (30 U/mL) for 30 min at 60 °C. The reaction solution was analyzed using ODS HPLC-MS (ESI (negative)) (HPLC conditions were the same as those used for the quantification analyses of total flavonoids), and a new peak (*t_R_* 12.0 min) with the same mass as C-3 rhamnose was eliminated from **4** (in this HPLC analysis, compound **4** was eluted at *t_R_* 11.3 min).

### 2.9. Determination of D-Glucuronic Acid in Compound ***8*** by D-Glucuronidase

Compound **8** (1.0 mg) was dissolved in 0.1 M AcOH-NaOH buffer (pH 5.0) (1 mL) and allowed to react with 1 mL of β D-glucuronidase from Helix pomatia (Funakoshi Co., Ltd., Tokyo, Japan) (105 U/mL) for 30 min at 60 °C. The reaction solution was analyzed using ODS HPLC-MS (ESI (negative)) (HPLC conditions were the same as those used for quantification analyses of total flavonoids) to obtain a new peak (*t_R_* 11.4 min) with the same mass as the C-3 rhamnose eliminated from **8** (in this HPLC analysis, compound **8** was eluted at *t_R_* 10.3 min).

### 2.10. Assessment of Lipid Peroxidation-Inhibiting Activity

The lipid peroxidation-inhibiting activity of the antioxidant compounds was assessed in brain homogenates as described previously [14]. Frozen rat brains were defrosted in ice-cold 0.1 M phosphate buffer (pH 7.2). Then, 0.8 g of each brain sample was mixed with 30 mL of ice-cold phosphate buffer for 2 min in a Teflon homogenizer. Next, 200 μL of the resulting homogenate, 0.65 mL 0.1 M phosphate buffer (pH 7.2) with or without 0.15 mM FeSO_4_, and 0.1 mL 1 mM sodium ascorbate were mixed with 50 μL of the test compound dissolved in MeOH (final concentration, 0.1–100 μM) and added to small glass test tubes (5 mL). The tubes were thoroughly agitated and incubated at 37 °C for 1 h, with reciprocal agitation.

To quantify thiobarbituric acid reactive substances production, the OD_532_ of the solution was measured. The inhibition of lipid peroxidation was assessed based on the IC_50_ value.

### 2.11. DPPH Radical-Scavenging Assay

The 2,2-Diphenyl-1-picrylhydrazyl (DPPH) radical-scavenging activity of each flavonoid compound was measured according to a previously described method [15], with some modifications. Briefly, 100 μL of the sample solution dissolved in EtOH (final concentration, 0.1–100 μM) was added to a 96-well plate, and 100 μL of the DPPH solution dissolved in EtOH (final concentration: 1 mM) was added to each well. The plates were incubated in the dark for 30 min at 20 °C. The change in absorption at 550 nm was measured using a multilabel counter.

### 2.12. Statistical Analysis

Data obtained from the lipid peroxidation-inhibiting and DPPH radical-scavenging assays were analyzed using one-way analysis of variance among subjects, and post hoc comparisons were made using the Student’s t test. In all cases, statistical significance was set at *p* < 0.05.

### 2.13. Physicochemical Data for Compounds ***1***–***8***

Physicochemical data for compounds **1**–**8** are available in Supplementary Materials (Physicochemical data S1–S8).

## 3. Results

### 3.1. Isolation of Antioxidative Compounds from the Flowers of Freesia ‘f2’ and ‘Kayak’

The freesia cultivars and wild species used in this study are exhibited in Figure 1. The ‘f2’ and ‘Kayak’ flowers were used to isolate compounds **1**–**6** and **7**, **8**, respectively. Both dried flowers from the cultivars ‘f2’ and ‘Kayak’ were extracted using CH_2_Cl_2_-MeOH (1:1), MeOH, and 50% (*v*/*v*) MeOH in a stepwise manner, and the extracts were tested using the lipid peroxidation-inhibiting assay, since the freesia apocarotenoids do not have this peroxidation-inhibiting activity [12]. Antioxidant activity was observed in MeOH and 50% MeOH extracts of both flowers. These extracts were concentrated to dryness in vacuo and further purified using preparative ODS HPLC. Eight pure compounds (**1**–**6** from ‘f2’ (Figure S1), **7** and **8** from ‘Kayak’ (Figure S2)) were obtained using preparative ODS HPLC.

### 3.2. Structural Determination of Compounds ***1***–***8***

The molecular formula of compound **1** was determined as C_42_H_46_O_24_ using HR-ESI-MS (positive) analysis (*m*/*z* 957.22643 (M + Na)^+^, calculated for 957.22767 (C_42_H_46_NaO_24_, Δ1.30 ppm))

Comparison of the observed ^1^H and ^13^C NMR spectra of **1** in DMSO-*d*_6_ (Figures S3 and S4) with those previously reported for quercetin and caffeic acid strongly suggested that 1 possesses quercetin and caffeic acid as its partial structure. Additionally, the presence of two hexoses and one 6-deoxyhexose in **1** was confirmed by the correlations observed in the 2D NMR spectra (^1^H-^1^H DQF COSY, HSQC, and HMBC).

**Figure 2:**
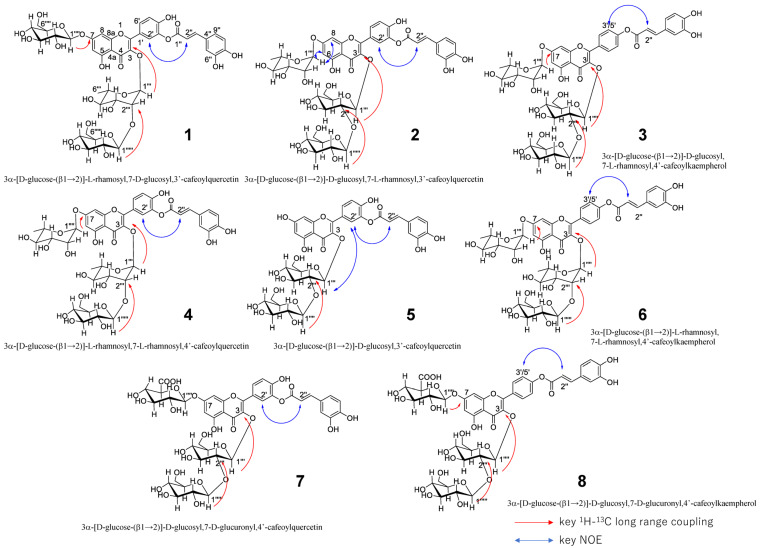
Structures of 3′-caffeoylquercetin glycosides and 4′-caffeoylkaempferol glycosides identified in the freesia petals.

The presence of quercetin and caffeic acid structures was confirmed using 2D NMR (^1^H-^1^H DQF COSY, HSQC, and HMBC) spectral analyses, and the ester linkage of caffeic acid to quercetin at C-3′ was proved by the observation of NOE between H-2′ (δ 7.39) and H-2″ (δ 6.17) in the NOESY spectrum (Figure 2).

Two hexoses (C-1′′′′–C-6′′′′ and C-1′′′′′–C-6′′′′′) in 1 were identified as both β-glucose using the analyses vicinal spin coupling constants of H-1′′′′–H-5′′′′ and H-1′′′′–H-5′′′′ (*J* = 6.2–8.5 Hz) elucidated using the J-resolved HSQC spectrum. One 6-deoxyhexose (C-1′′′–C-6′′′) in 1 was identified as α-rhamnose using the analyses of vicinal spin coupling constants of (*J*_1,2_ = 0 Hz, *J*_2,3_ = 2.0 Hz, *J*_3,4_ = 9.0 Hz, *J*_4,5_ = 7.8 Hz) and the large JCH coupling constant of C-1′′′ (JCH = 175 Hz) [16].

The linkage of one glucose (C-1′′′′′–C-6′′′′′) to α-rhamnose at C-2′′′ was proven by the ^1^H–^13^C long-range coupling from H-1′′′′′ (δ 4.26) to C-2′′′ (δ 81.7) and NOE between H-1′′′′′ and H-2′′′ (δ 4.13), and the linkage of the other glucose (C-1′′′′–C-6′′′′) to quercetin at C-7 was proven using the ^1^H–^13^C long-range coupling from H-1′′′′ (δ 5.25) to C-7 (δ 162.7) and NOEs between H-1′′′′ and H-6 (δ 6.42) and H-1′′′′ and H-8 (δ 6.72) (Figure 2). The linkage of α-rhamnose to quercetin at C-3 was also proven by ^1^H–^13^C long-range coupling from H-1′′′ (δ 5.49) to C-3 (δ 134.7) (Figure 2). From these observations, the structure of compound **1** (3α-[glucose-(β1 → 2)]-rhamosyl,7-glucosyl,3′-cafeoylquercetin) was clarified as shown in Figure 2.

The structures of compounds **2**–**8** were analyzed using HR-ESI-MS (positive or negative) and 1D (^1^H and ^13^C) and 2D NMR (^1^H-^1^H DQF COSY, HSQC, *J*-resolved HSQC, HMBC, and NOESY) analyses like compound **1** and determined as shown in Figure 2. The observed key ^1^H–^13^C long-range couplings and NOEs observed for each compound are shown in Figure 2. ^1^H and ^13^C NMR spectra of compounds **2**–**8** are shown in Figures S5–S18.

To determine the absolute configuration of glucose in compounds **1**–**8**, compound **5** was hydrolyzed in 2.0 N HCl. The solution was partitioned between EtOAc/H_2_O, and the H_2_O layer containing glucose was further purified using silica gel column chromatography (CH_2_Cl_2_:MeOH (2:1)) to obtain pure glucose. As the [α]_D_ value of this compound was + 46.4° (*c* 0.2), it was identified as D-glucose.

To assign the absolute configuration of rhamnose in compounds **1**–**4** and **6**, compound **4** (1.0 mg) was dissolved in 0.1 M phosphate buffer (pH 7.5) and allowed to react with α-L-rhamnosidase (Funakoshi Co., Ltd., Tokyo, Japan) for 30 min at 60 °C. The reaction solution was analyzed using ODS HPLC-MS, and a new peak with the same mass as that of C-3 rhamnose, eliminated from **4** (Figure S19), was observed in the chromatogram. Thus, the rhamnose moiety in compound **4** was confirmed to be L-rhamnose.

To assign the absolute configuration of glucuronic acid in compounds **7** and **8**, compound **8** (1.0 mg) was dissolved in 0.1 M AcOH-NaOH buffer (pH 5.0) and allowed to react with β-D-glucuronidase (Funakoshi Co., Ltd., Tokyo, Japan) for 30 min at 60 °C. The reaction solution was analyzed using ODS HPLC-PDA-MS, and a new peak with the same mass as C-3 glucuronic acid eliminated from **8** (Figure S20) was observed in this chromatography. Therefore, the glucuronic acid in compound **8** was confirmed as D-glucuronic acid. Based on the observations described above, the absolute structures of 1–8 were determined, as shown in Figure 2.

### 3.3. Analysis of Flavonoid Compounds in the Petals of Individual Freesia Species

Figure S21 shows HPLC-PDA profiles of the extracts from the petals of yellow-flower freesia cultivars ‘f2’, ‘Kayak’, ‘Passat’, and ‘Boulevard’. As shown in this Figure, ‘f2’ included a few peaks of unidentified flavonoid glycosides and compounds **1**–**6**.

Compounds **7** and **8**, flavonoids with 7-O-glucuronic acid, were observed in ‘Kayak’, ‘Passat’, and ‘Boulevard’ as the dominant flavonoids and not observed in ‘f2.’ We next conducted HPLC-PDA analyses on the extracts from the petals of white-flower freesia cultivars ‘Silk’ and ‘White’ and freesia wild species ‘Alba’ (white-flower species) and *F. refracta*. The results are shown in Figure S22. These petals accumulated several flavonoid glycosides, including compound **2** (and **3**) in common. Compounds **7** and **8** were not observed in white petals, including those of *F. refracta*. Flavonoid glycosides are also present in freesia white petals, regardless of the cultivar or wild species.

The approximate amount of total flavonoid compounds in the petals of individual freesia species was calculated from the peak areas at 330 nm, as shown in Figure 1. Cultivars ‘Kayak’ and ‘Passat’ produced the highest amounts of the flavonoid glycosides.

### 3.4. Antioxidant Activities of Compounds ***1***–***8***

We examined the antioxidant activity of compounds **1**–**8** by inhibiting lipid peroxidation and quenching 2,2-diphenyl-1-picrylhydrazyl (DPPH) radicals. The results are summarized in Table 1. Compounds **1**–**8** possessed antioxidant activity.

## 4. Discussion

We discovered eight novel flavonoids (polyphenols) by their structural determination in the yellow petals of yellow-flower freesia. Four 3′-caffeoylquercetin 3,7-glycosides (compounds **1**, **2**, **4**, and **7**), one 3′-caffeoylquercetin 3-glycoside (3-sophoroside; compound **5**), and three 4′-caffeoylkaempferol 3,7-glycosides (compounds **3**, **6**, and **8**) were identified. The C-3 sugar part was not a major glucose monomer but a minor hexose dimer [β-D-glucopyranosyl-(1,2)-D-glucopyranoside (sophoroside) or β-D-glucopyranosyl-(1,2)-α-L-rhamnopyranoside], while the C-7 were usually O-glycosylated with D-glucose, D-glucuronic acid, or L-rhamnose.

Flavonol glycosides, including a disaccharide that is O-linked to the C-3 position and does not contain caffeic acid, have been found in many higher plants including edible flowers. For example, quercetin 3-sophoroside and kaempferol 3-sophoroside have been identified in flowers of the subgenus Rosa [17]. The leaves of *Ginkgo biloba* were found to retain quercetin 3-O-β-D-glucosyl-(1,2)-α-L-rhamnoside, which included the same sugar structure as that of freesia [18]. In contrast, rutin [quercetin 3-O-β-rutinoside, i.e., quercetin 3-O-α-L-rhamnosyl-(1,6)-β-D-glucoside], known as one of representative Citrus flavonol glycosides, showed a distinct structure from that of freesia [19].

**Figure 3:**
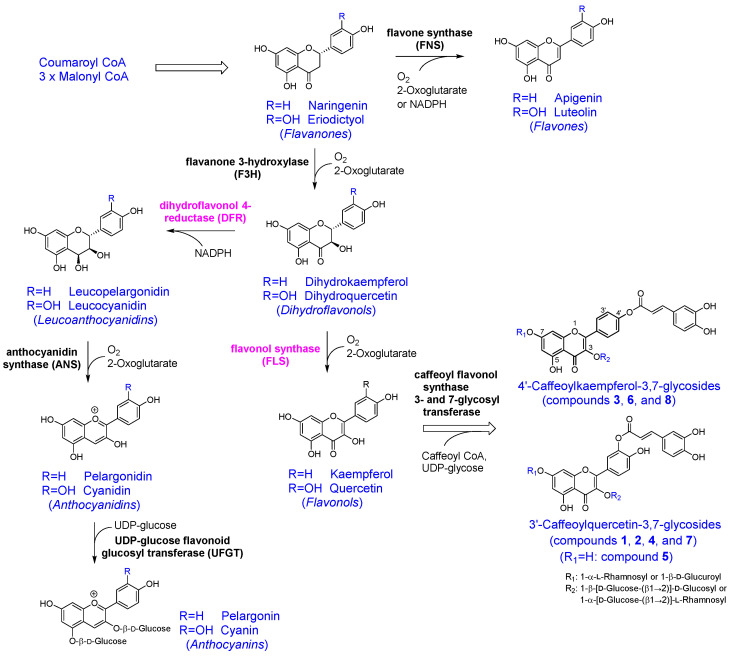
Pathways of flavonoids (anthocyanins, flavones, and flavonols, and feasible route for compounds **1**–**8** from flavonols) in the differentiating tissues from floral buds to petals of the yellow-flower freesia cultivars. Box arrows represent multiple enzyme reactions. Conversion of flavonoid (R=H; e.g., naringenin) to flavonoid (R=OH; e.g., eriodictyol) is catalyzed by flavonoid 3′-hydroxylase (F3′H).

The biosynthetic pathway of flavonoids (anthocyanins, flavones, and flavonols) in freesia petals (including tissues differentiating to petals) is shown in Figure 3, which further includes a feasible biosynthetic route for compounds **1**–**8** from flavonols. Uridine diphosphate-glycose (UDP-glycose), flavonoid 3-O-glycosyltransferase (3-glycosyl transferase; UF3GT), and 7-glycosyltransferase (UF7GT) are considered to be enzymes that mediate the biosynthesis of compounds **1**–**8** from their aglycons. Although these genes remain unknown, freesia has been reported to possess several paralogs of the UF3GT gene [20]. Yellow-flower freesia additionally produces crocetin esterified with the neapolitanosyl group, which includes both β1,2- and β1,6-linkage of D-glucose [12]. Freesia is likely to be an excellent source of glycosyltransferase genes.

Flavonols and anthocyanins share biosynthetic pathways that produce dihydroflavonols, which are the last common precursors between them and branches off [1,2,11] (Figure 3). Dihydroflavonol 4-reductase (DFR) is a key enzyme that catalyzes the reduction of dihydroflavonols to leucoanthocyanidins for the biosynthesis of anthocyanins with pink, blue, and purple colors. Freesia was reported to retain diverged *DFR* (*FhDFR*) or *DFR*-like genes [11]. It was further shown that the individual introduction of several *FhDFR* genes into *Arabidopsis dfr* (*tt3-1*) mutant plants resulted in partial complementation of the loss of cyanidin derivative synthesis, indicating that the FhDFR enzymes can convert dihydroquercetin to leucocyanidin [11]. On the other hand, flavonol synthase (FLS) is required to biosynthesize compounds **1**–**8** via flavonol, quercetin, or kaempferol. These results imply that, when the *DFR* (*FhDFR*) genes are not expressed and instead the *FLS* gene is functionally expressed in freesia flowers and floral relevant organs, the produced dihydroflavonols could switch to the stream for flavonol formation. The aglycones of compounds **1**–**8** were likely generated by an esterification reaction between quercetin or kaempferol and caffeoyl-CoA (Figure 3). The existence of such caffeoyl-flavonol glycosides (final products) in the petals likely supports the generation of bright white and yellow colors according to the absence and presence of the apocarotenoids, neapolitanosyl crocetin and dineapolitanosyl crocetin.

Indeed, there are no other reports that have written of the presence of natural compounds in which flavonol (quercetin or kaempferol) and caffeic acid are directly esterified while synthetic compounds including caffeoylquercetin in the molecules, e.g., tri(diacetylcaffeoyl) quercetin, have been made to examine their antioxidant activities [21]. We evaluated the lipid peroxidation-inhibiting activities of compounds **1**–**8**, as shown in Table 1. All the compounds showed potent lipid peroxidation-inhibitory activities (IC_50_ 0.63–27 μM). Concerning this activity, the compounds including the quercetin aglycone (**1**, **2**, **4**, **5**, and **7**; IC_50_ 0.63–5.2 μM) in the molecules were superior to those including the kaempferol aglycone (**3**, **6**, and **8**; IC_50_ 20–27 μM) (Table 1). Thus, phenolic OH function in the flavone C ring (C-3′) may be important for the quenching activity. The antioxidant activities of compounds **1**–**8** were further examined by determining their DPPH radical-scavenging activities (Table 1). All the compounds retained their antiradical activity. Among them, compounds **5** and **7**, including quercetin aglycone, showed the most potent DPPH radical-quenching activities (IC_50_ = 12 and 10 μM, respectively), which were superior to those of quercetin, caffeic acid, and rutin (IC_50_ = 23, 41, and 14 μM, respectively). Compounds **5** and **7** were found to exert superior antioxidant activity, not only for quenching DPPH radicals but also for inhibiting lipid peroxidation, compared with rutin, a known typical quercetin 3-glycoside. In particular, compound **5** exceeded rutin significantly in lipid peroxidation-inhibiting activity.

As for edible flowers, the petals have been shown to be a source of bioactive phenolic compounds including polyphenols, while the presence of quercetin and kaempferol glycosides has been reported to be a key for potent antioxidant activities [6,17,22,23]. Pharmaceutical and preventive activities of quercetin, kaempferol, and caffeic acid against cardiovascular diseases, inflammatory diseases, and diabetes have also been reported [23,24,25]. Compounds **1**–**8** could be new ingredients in food to prevent these diseases.

## 5. Conclusions

The existence of antioxidant chemicals unique to petals was demonstrated in the flowering plant freesia. These compounds were glycosides of 3′-caffeoylquercetin and 4′-caffeoylkaempferol, i.e., two polyphenol aglycons with new structures in the natural world. When dihydroflavonols enter the route for flavonol synthesis, these caffeoyl-flavonol glycosides are likely to be biosynthesized via quercetin or kaempferol to support the generation of bright white and yellow colors with and without the presence of the apocarotenoids, respectively. All the identified caffeoyl-flavonol glycosides exerted in vitro antioxidant activities against both lipid peroxidation and radical generation. Specifically, the flavonoid glycosides including quercetin such as 3′-caffeoylquercetin 3-sophoroside and its 7-glucuronide showed superior antioxidant activity to those including kaempferol and to rutin. Yellow and white freesia flowers have also been utilized as edible flowers, indicating the importance of evaluating the human benefits and risks of newly identified flavonoids.

## Figures and Tables

**Table 1:** Antioxidant activities of individual compounds.

Compound	IC_50_ (μM)	IC_50_ (μM)
	**Lipid Peroxidation-Inhibiting Activity**	**DPPH Radical-Quenching Activity**
**1**	5.2 ± 0.62	32 ± 2.2
**2**	4.5 ± 0.73	29 ± 2.0
**3**	22 ± 0.96	38 ± 2.2
**4**	1.9 ± 0.21	22 ± 4.4
**5**	0.63 ± 0.04	12 ± 0.77
**6**	20 ± 1.7	56 ± 5.1
**7**	1.1 ± 0.82	10 ± 0.54
**8**	27 ± 3.1	16 ± 2.2
Quercetin	0.45 ± 0.04	23 ± 0.89
Caffeic acid	25 ± 2.7	41 ± 0.52
Rutin	11 ± 0.82	14 ± 0.87

Yellow backgrounds indicate compounds including the quercetin structure in the molecules.

## Data Availability

All data generated in this study are included in the article and its Supplementary Materials.

## Supplementary Materials

The following supporting information can be downloaded at: https://www.mdpi.com/article/10.3390/antiox14020158/s1, Figure S1: Preparative HPLC-PDA profile of compounds **1**–**6** in the extract of the ‘f2’ flowers, Figure S2: Preparative HPLC-PDA profile of compounds **7** and **8** in the extract of the ‘Kayak’ flowers, Figure S3: 400 MHz ^1^H NMR spectrum of compound **1** in DMSO-*d*_6_, Figure S4: 100 MHz ^13^C NMR spectrum of compound **1** in DMSO-*d*_6_, Figure S5: 400 MHz ^1^H NMR spectrum of compound **2** in DMSO-*d*_6_, Figure S6: 100 MHz ^13^C NMR spectrum of compound **2** in DMSO-*d*_6_, Figure S7: 400 MHz ^1^H NMR spectrum of compound **3** in DMSO-*d*_6_, Figure S8: 100 MHz ^13^C NMR spectrum of compound **3** in DMSO-*d*_6_, Figure S9: 400 MHz ^1^H NMR spectrum of compound **4** in DMSO-*d*_6_, Figure S10: 100 MHz ^13^C NMR spectrum of compound **4** in DMSO-*d*_6_, Figure S11: 400 MHz ^1^H NMR spectrum of compound **5** in DMSO-*d*_6_, Figure S12: 100 MHz ^13^C NMR spectrum of compound **5** in DMSO-*d*_6_, Figure S13: 400 MHz ^1^H NMR spectrum of compound **6** in DMSO-*d*_6_, Figure S14: 100 MHz ^13^C NMR spectrum of compound **6** in DMSO-*d*_6_, Figure S15: 400 MHz ^1^H NMR spectrum of compound **7** in DMSO-*d*_6_, Figure S16: 100 MHz ^13^C NMR spectrum of compound **7** in DMSO-*d*_6_, Figure S17: 400 MHz ^1^H NMR spectrum of compound **8** in DMSO-*d*_6_, Figure S18: 100 MHz ^13^C NMR spectrum of compound **8** in DMSO-d6, Figure S19: HPLC-PDA analysis of the reaction mixture of compound **4** with α-L-rhamosidase, Figure S20: HPLC-PDA analysis of the reaction mixture of compound **8** with β-D-glucuronidase, Figure S21: HPLC-PDA analysis of the extracts from the petals of the cultivars ‘f2’, ‘Kayak’, ‘Passat’, and ‘Boulevard’, Figure S22: HPLC-PDA analysis of the extracts from the petals of cultivars ‘f2’, ‘Silk’ and ‘White’, and wild species ‘Alba’ and *F. refracta*. Physicochemical data S1: Physicochemical data for compound **1**. Physicochemical data S2: Physicochemical data for compound **2**. Physicochemical data S3: Physicochemical data for compound **3**. Physicochemical data S4: Physicochemical data for compound **4**. Physicochemical data S5: Physicochemical data for compound **5**. Physicochemical data S6: Physicochemical data for compound **6**. Physicochemical data S7: Physicochemical data for compound **7**. Physicochemical data S8: Physicochemical data for compound **8**.
