# Supplementary material for: 3′-Caffeoylquercetin Glycosides and 4′-Caffeoylkaempferol Glycosides—Novel Antioxidant Flavonoids Discovered in the Freesia Yellow Flowers
Source: Antioxidants (Basel). 2025 Jan 28;14(2):158. doi: 10.3390/antiox14020158 (PMC11851390; doi:10.3390/antiox14020158)
Supplement: Supplementary file 1 [file antioxidants-14-00158-s001.zip › antioxidants-3403855-supplementary.pdf]

Supporting Materials

3’-Caffeoylquercetin Glycosides and 4’-Caffeoylkaempferol Glycosides – Novel Antioxidant Flavonoids Discovered in the Yellow Freesia Flowers

Kazutoshi Shindo[1], Nozomi Iwamoto[1], Mayu Ysami[1], Ayuna Saito[1], Miho Sato[1], Maho Sugaya[1], Nao Miyashita[2], Minoru Murahama[2], Yasuki Higashimura[3], Miho Takemura[4], Kazuo Furihata[5], Norihiko Misawa[4,6]

AUTHOR ADDRESS

1 Department of Food and Nutrition, Japan Women’s University, 2-8-1 Mejirodai, Bunkyo-ku, Tokyo 112-8681, Japan

2 Ishikawa Agriculture and Forestry Research Center, 295-1 Saida-machi, Kanazawa, Ishikawa 920-3198, Japan

3 Department of Food Science, Ishikawa Prefectural University, 1-308 Suematsu, Nonoichi-shi, Ishikawa 921-8836, Japan

4 Research Institute for Bioresources and Biotechnology, Ishikawa Prefectural University, 1-308 Suematsu, Nonoichi-shi, Ishikawa 921-8836, Japan

5 Division of Agriculture and Agricultural Life Sciences, The University of Tokyo, 1-1-1, Yayoi, Bunkyo-ku, Tokyo 113-8657, Japan.

6 Department of Nutrition, Hokuriku Gakuin University, 11 Mitsukoji, Kanazawa, Ishikawa 920-1396, Japan

Figure S1. Preparative HPLC-PDA profile of compounds **1** - **6** in the extract of the ‘f2’ flowers

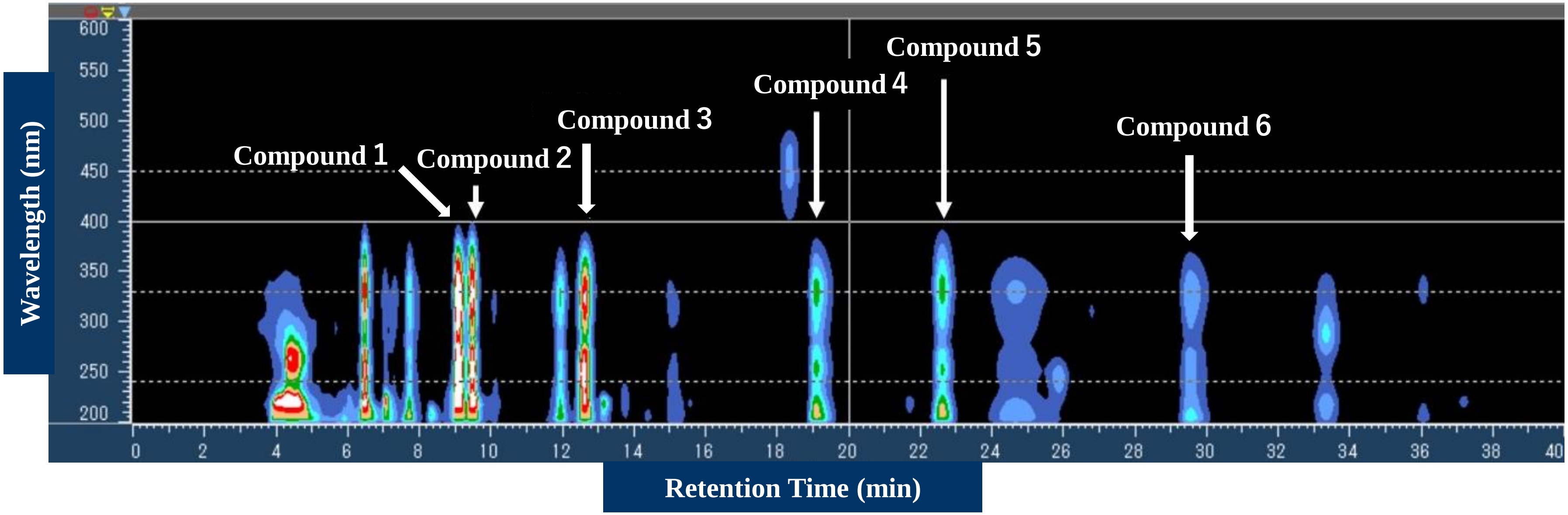

Figure S2. Preparative HPLC-PDA profile of compounds **7** and **8** in the extract of the ‘Kayak’ flowers

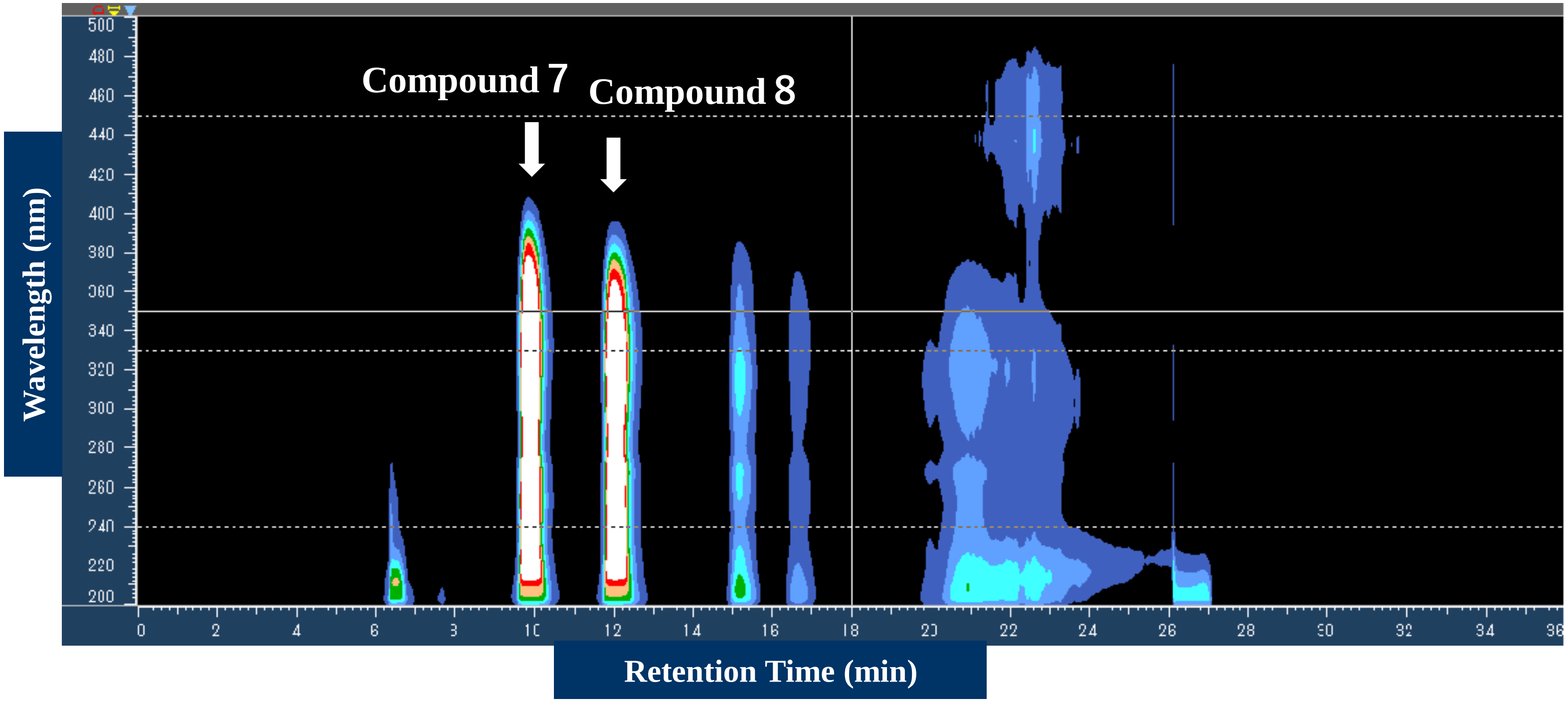

Figure S3. 400 MHz ^{1}H NMR spectrum of compound **1** in DMSO-d_6

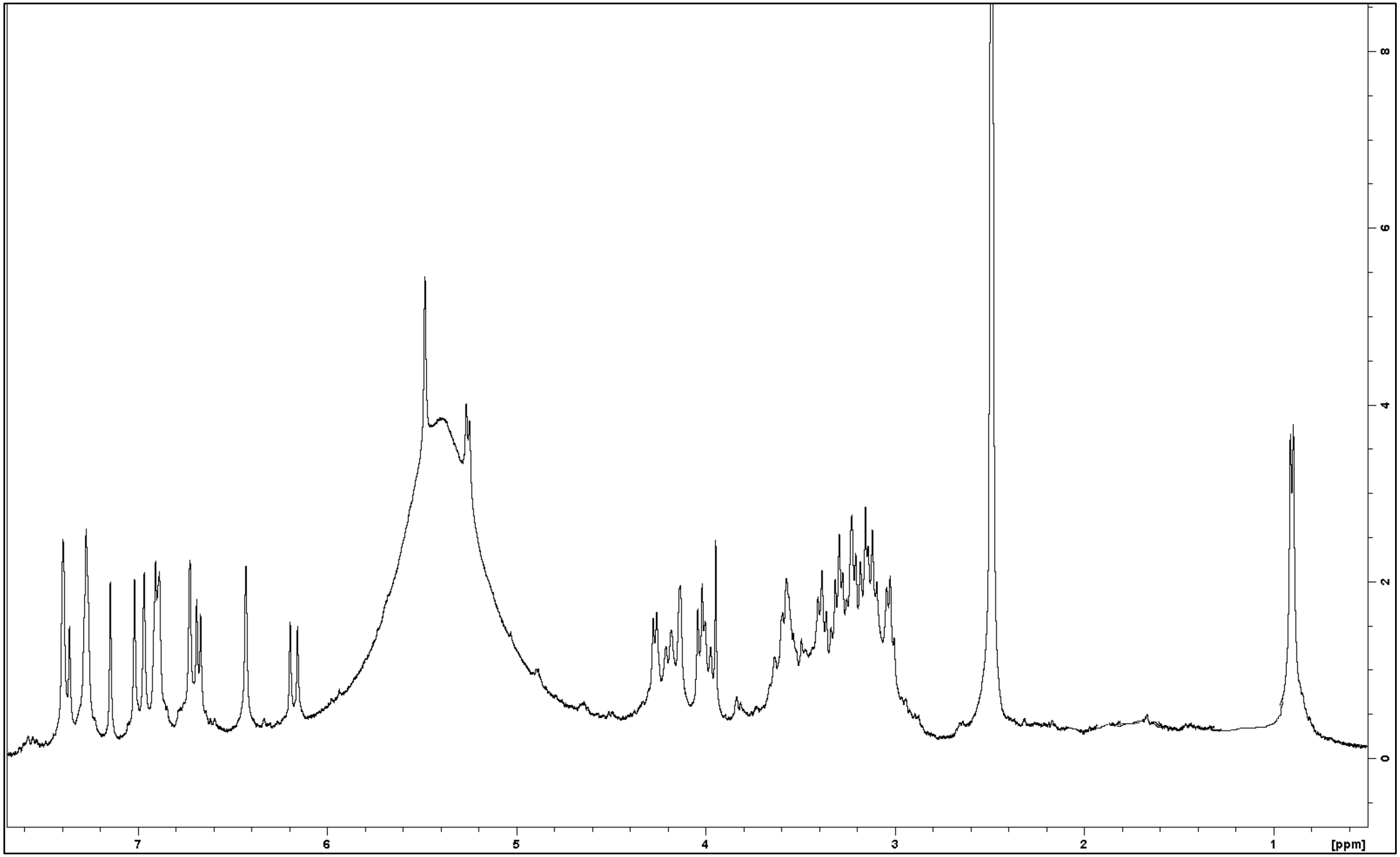

Figure S4. 100 MHz ^{13}C NMR spectrum of compound **1** in DMSO-d_6

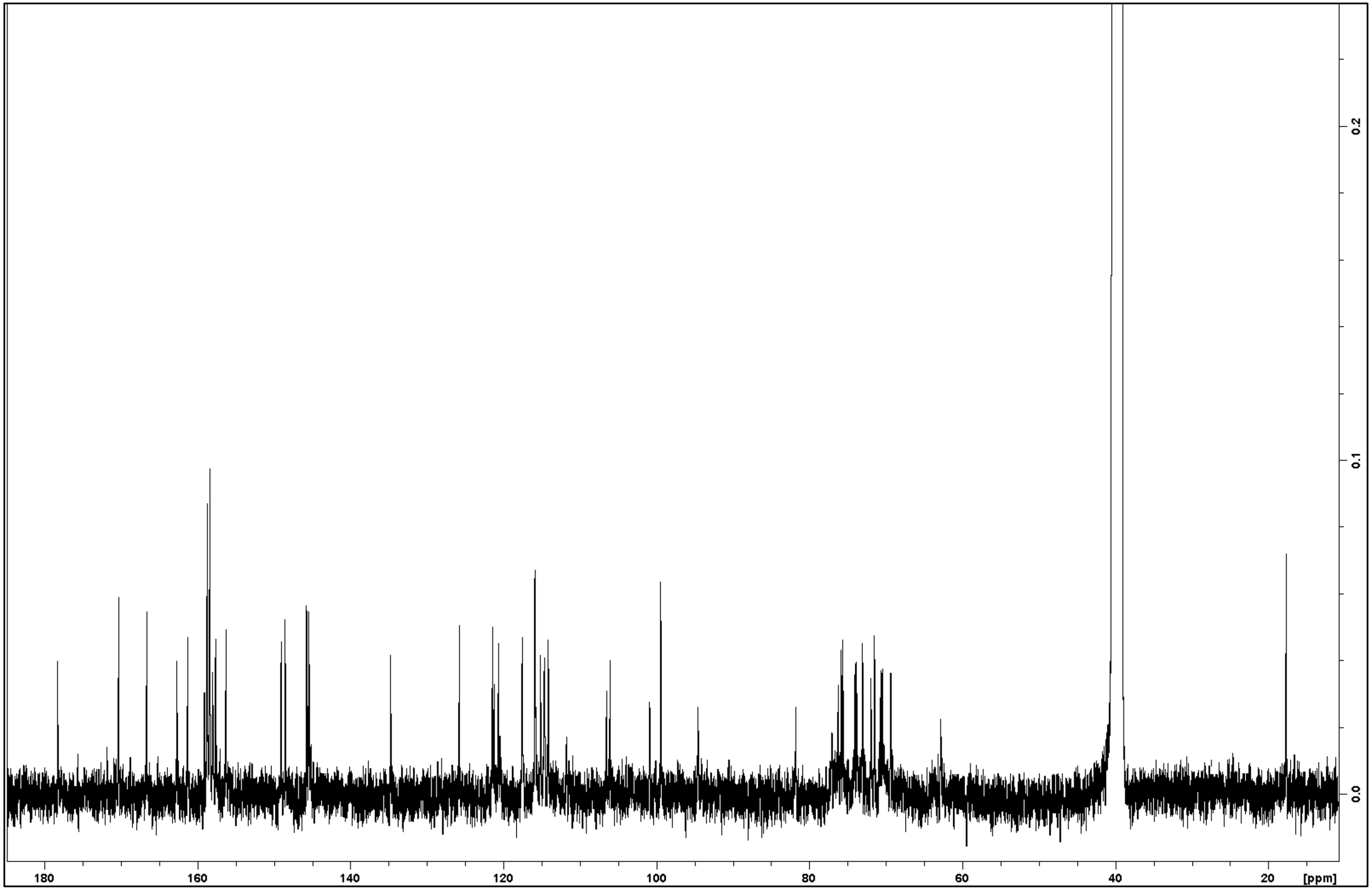

Figure S5. 400 MHz ^{1}H NMR spectrum of compound **2** in DMSO-d_6

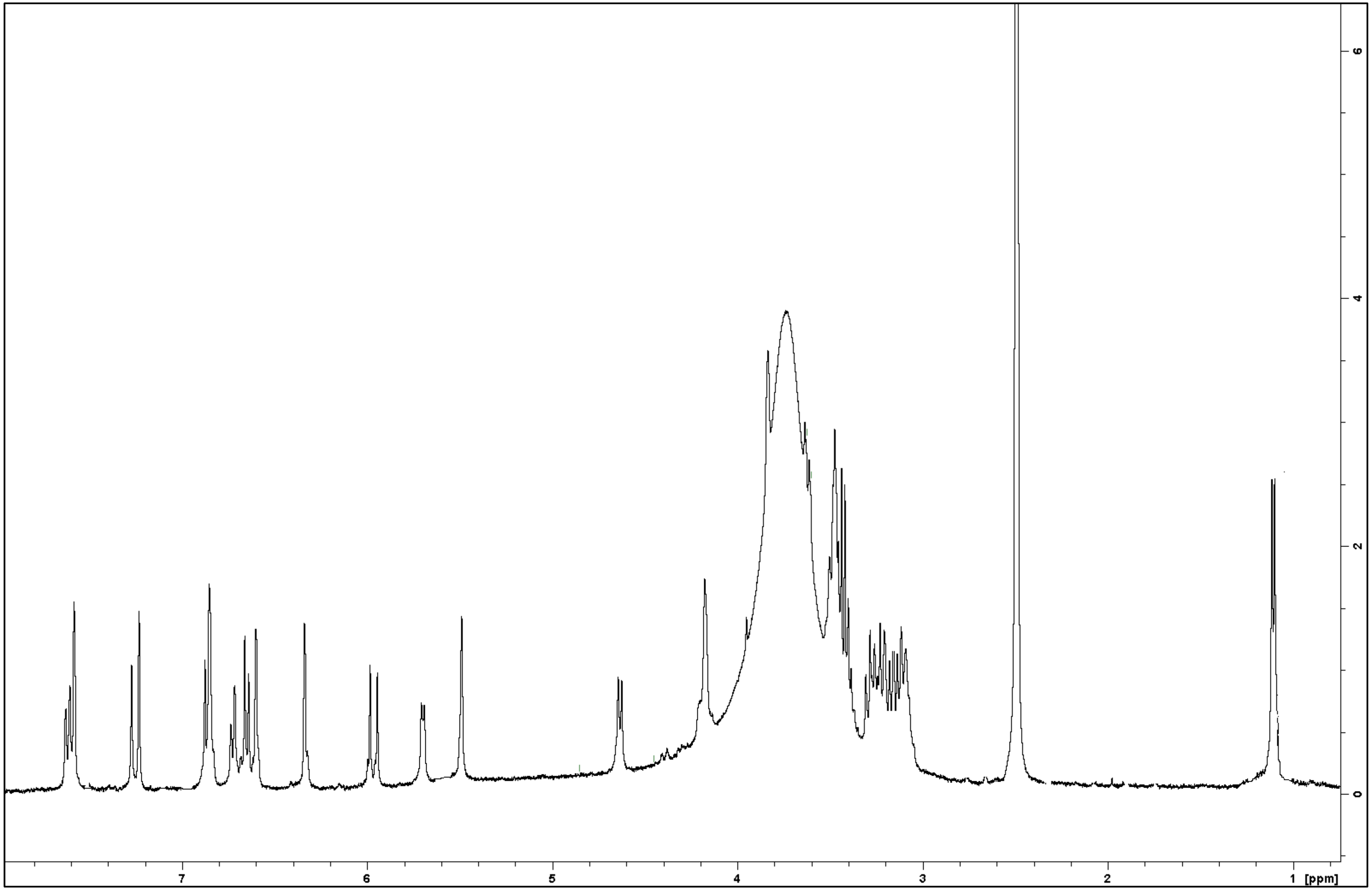

Figure S6. 100 MHz ^{13}C NMR spectrum of compound **2** in DMSO-d_6

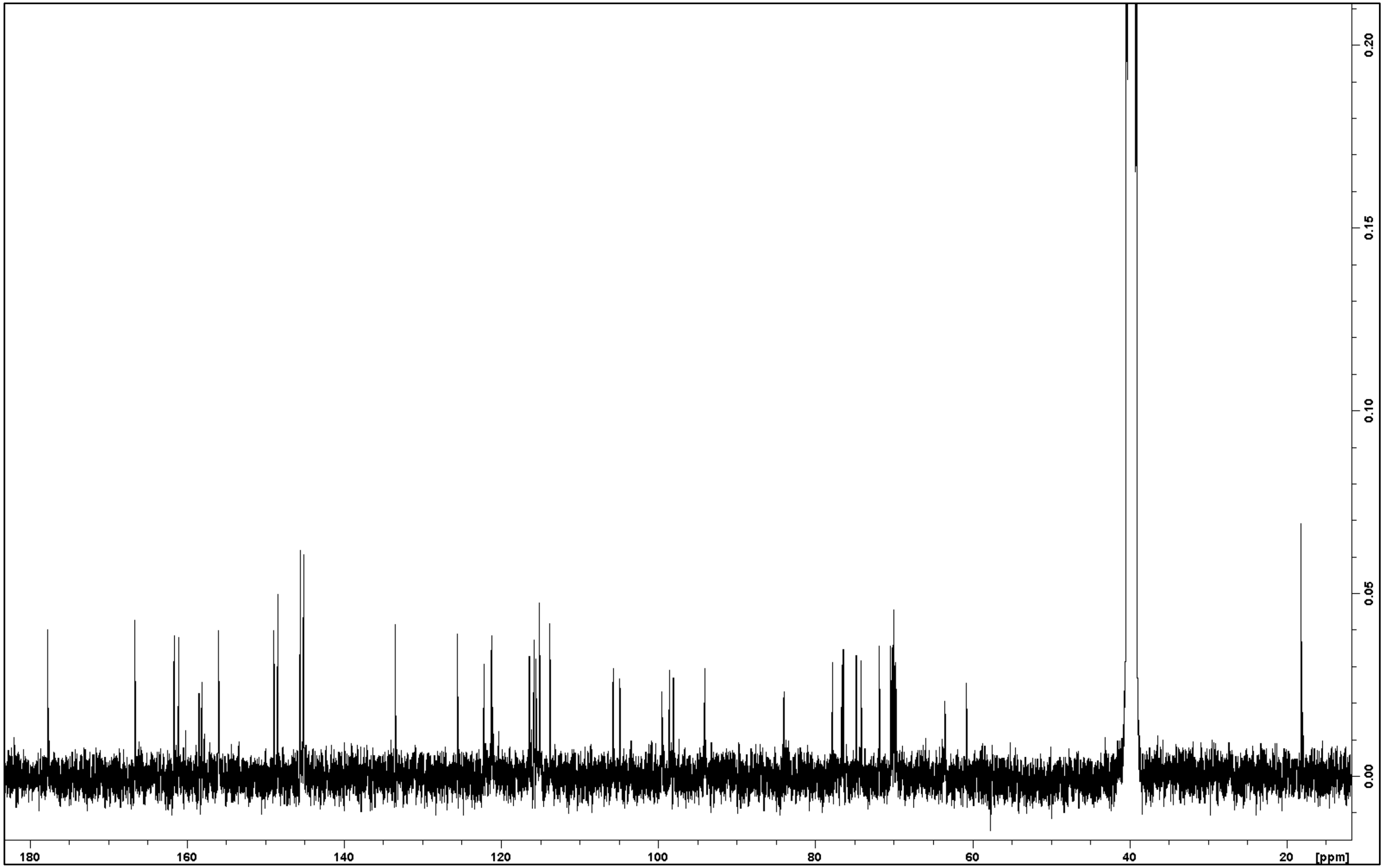

Figure S7. 400 MHz ^{1}H NMR spectrum of compound **3** in DMSO-d_6

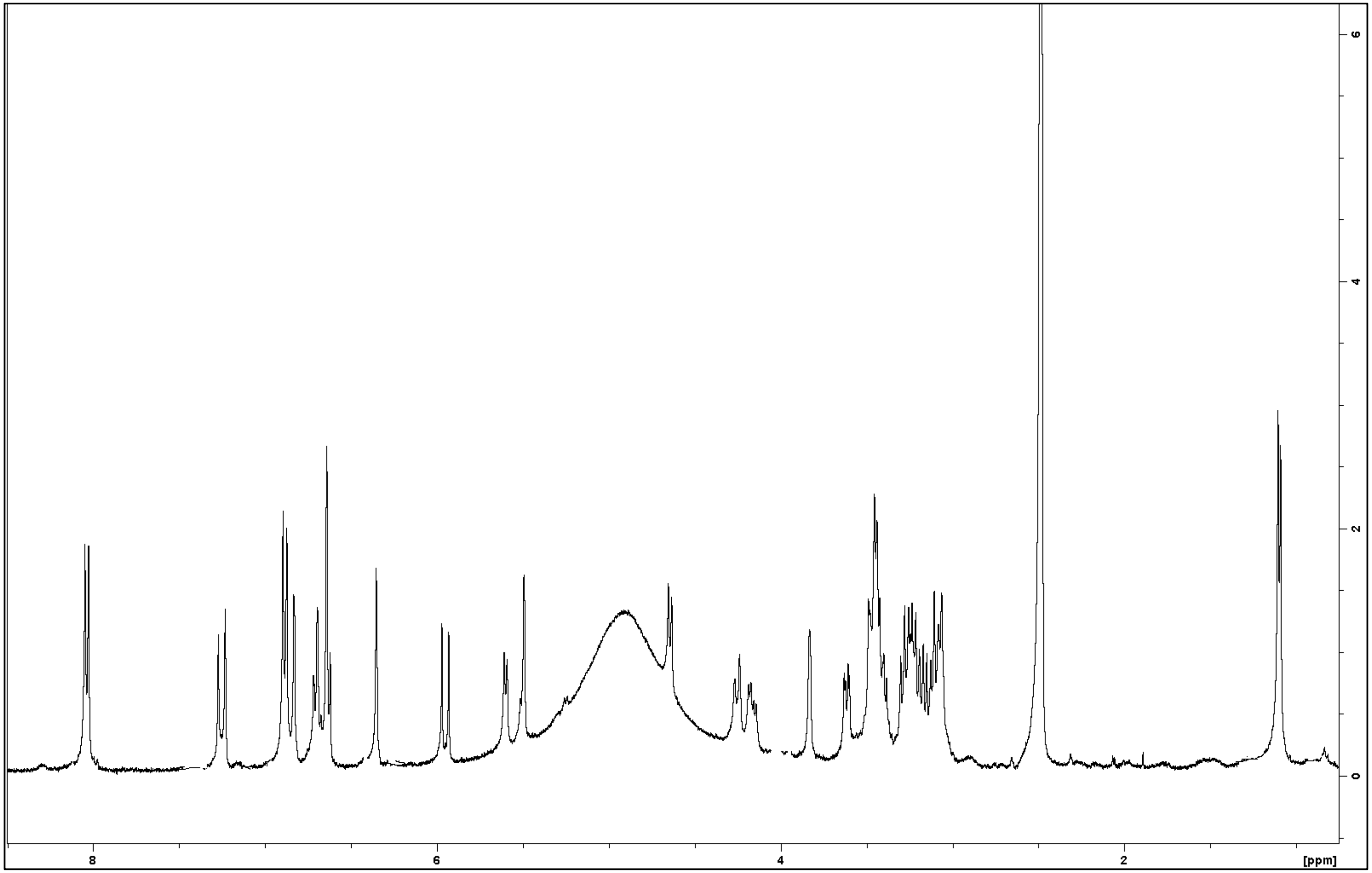

Figure S8. 100 MHz ^{13}C NMR spectrum of compound **3** in DMSO-d_6

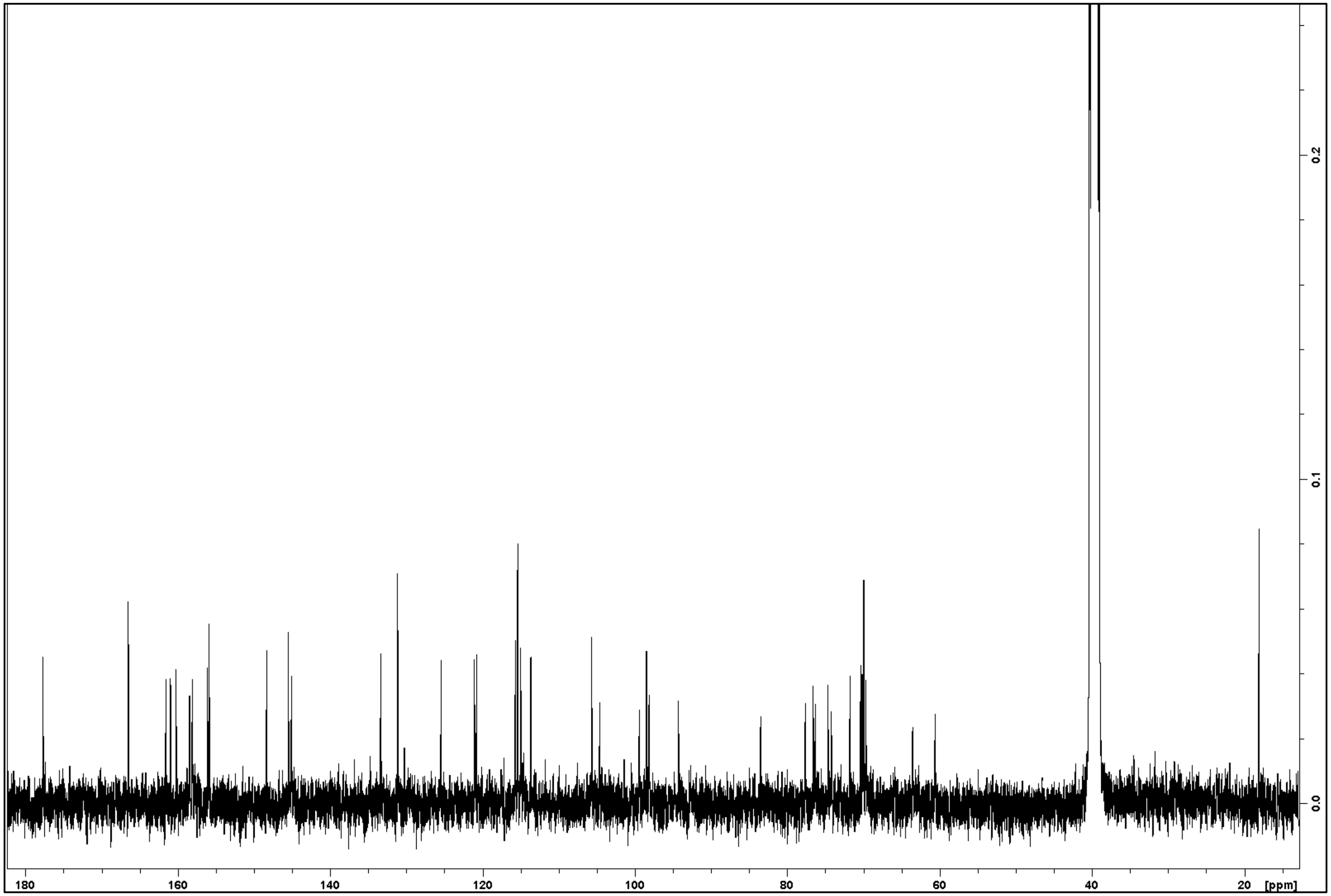

Figure S9. 400 MHz ^{1}H NMR spectrum of compound **4** in DMSO-d_6

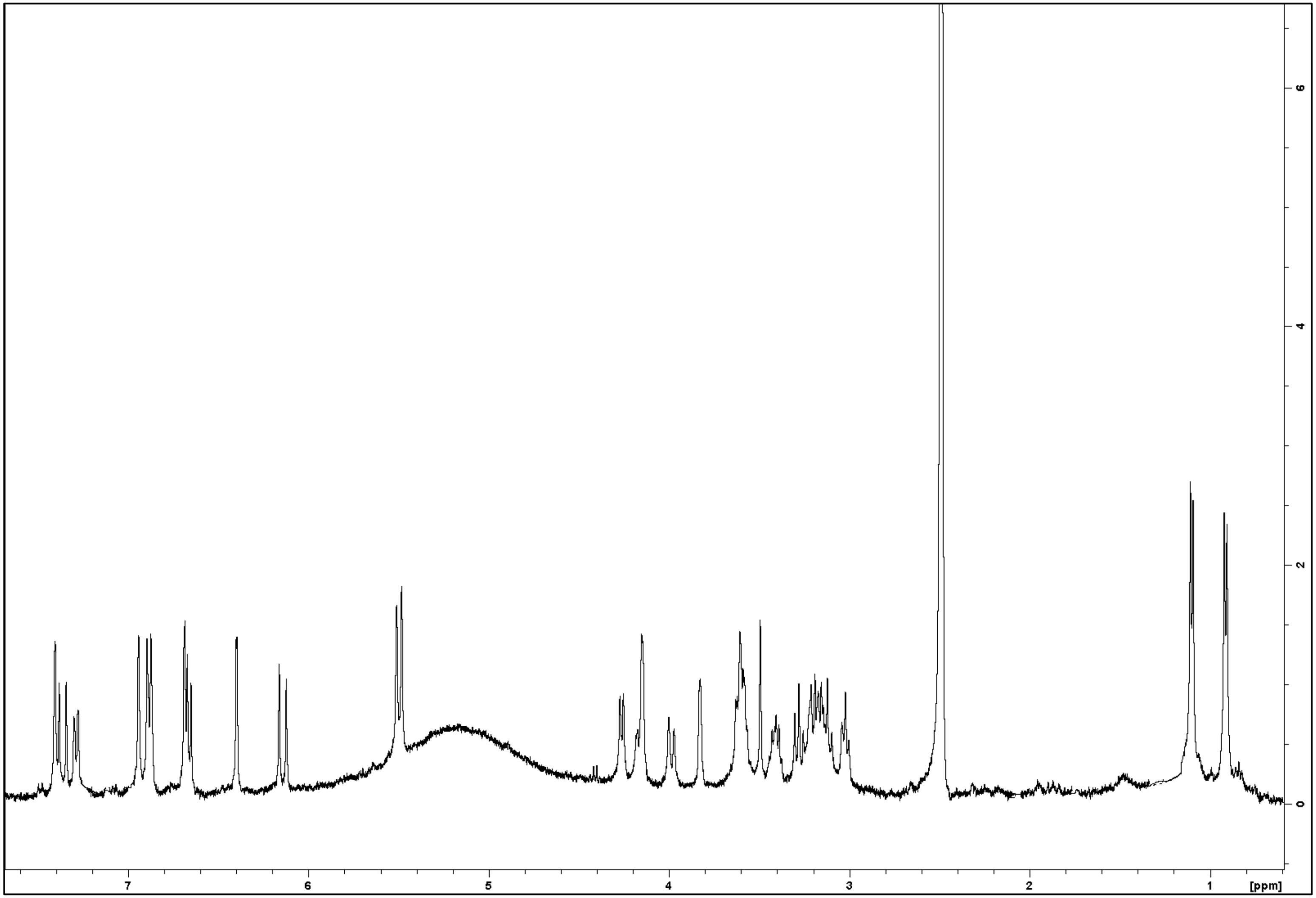

Figure S10. 100 MHz ^{13}C NMR spectrum of compound **4** in DMSO-d_6

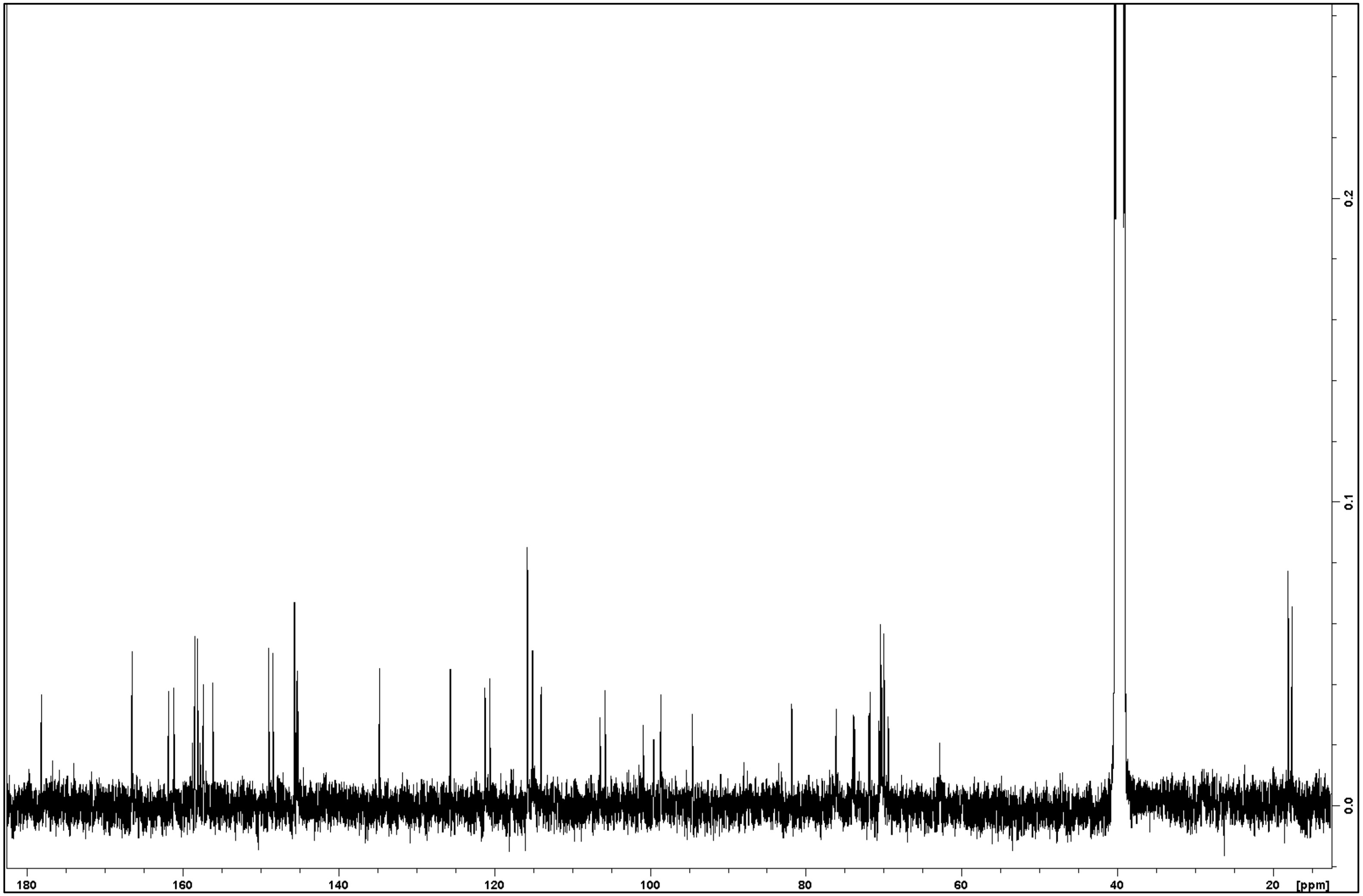

Figure S11. 400 MHz ^{1}H NMR spectrum of compound **5** in DMSO-d_6

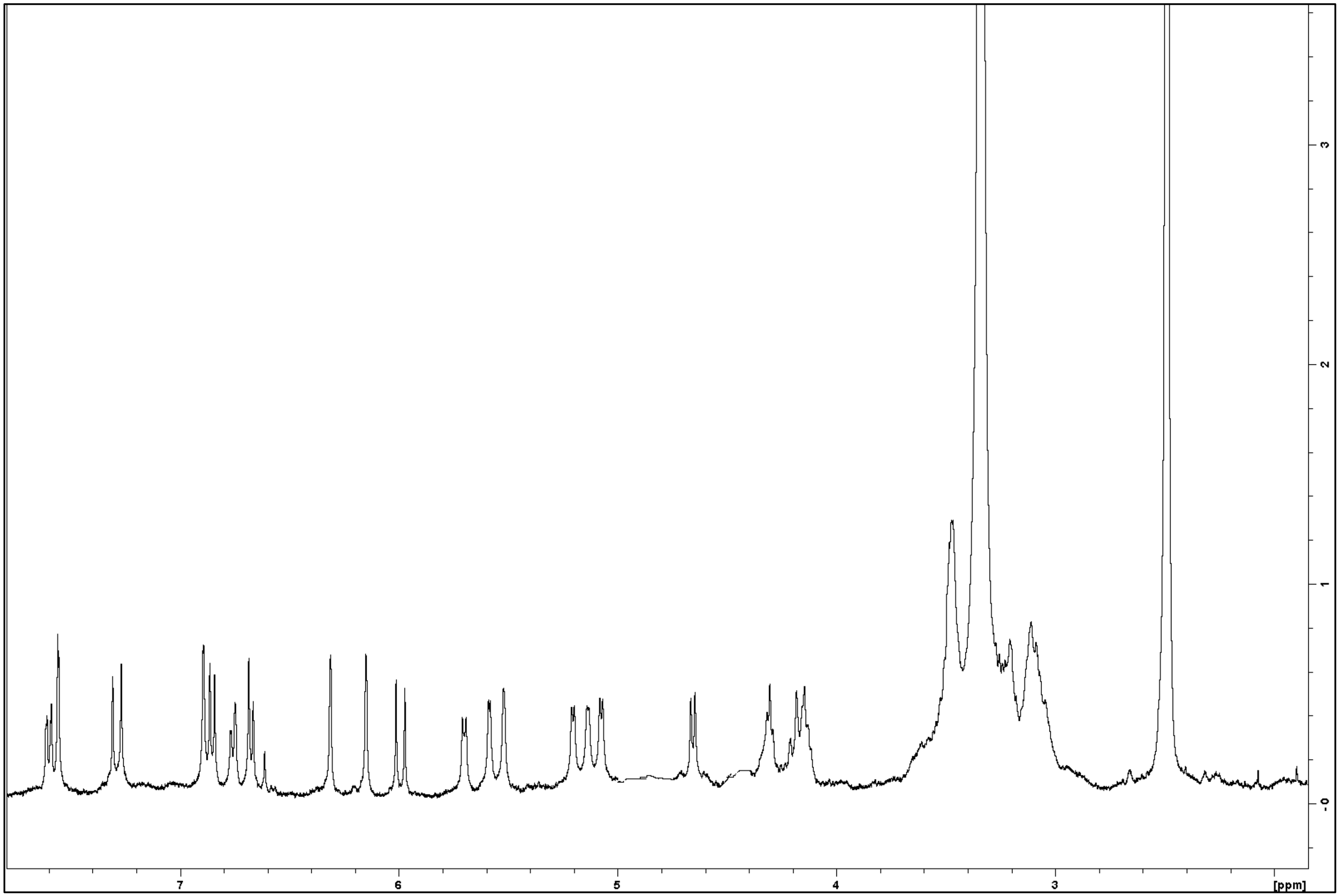

Figure S12. 100 MHz ^{13}C NMR spectrum of compound **5** in DMSO-d_6

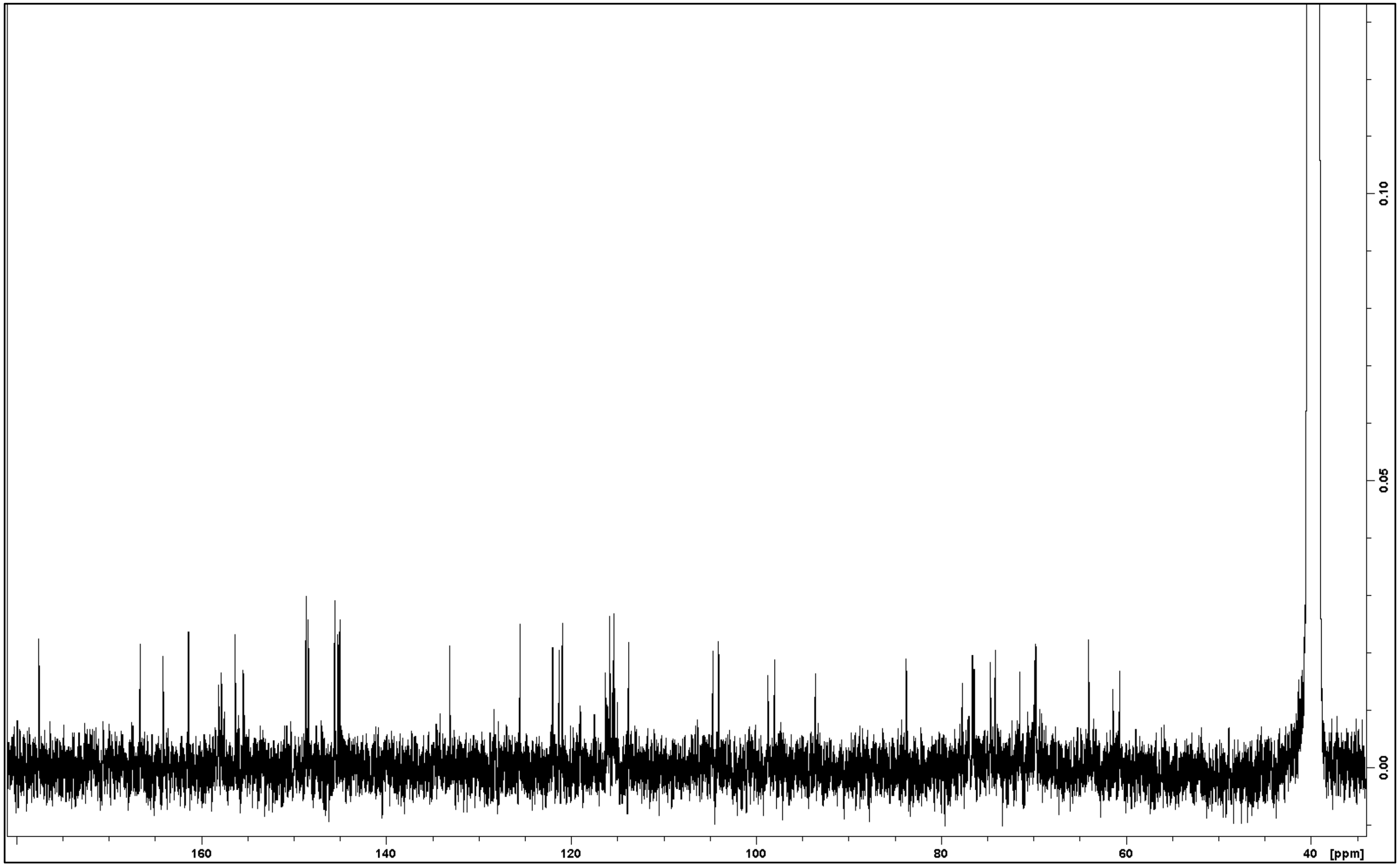

Figure S13. 400 MHz ^{1}H NMR spectrum of compound **6** in DMSO-d_6

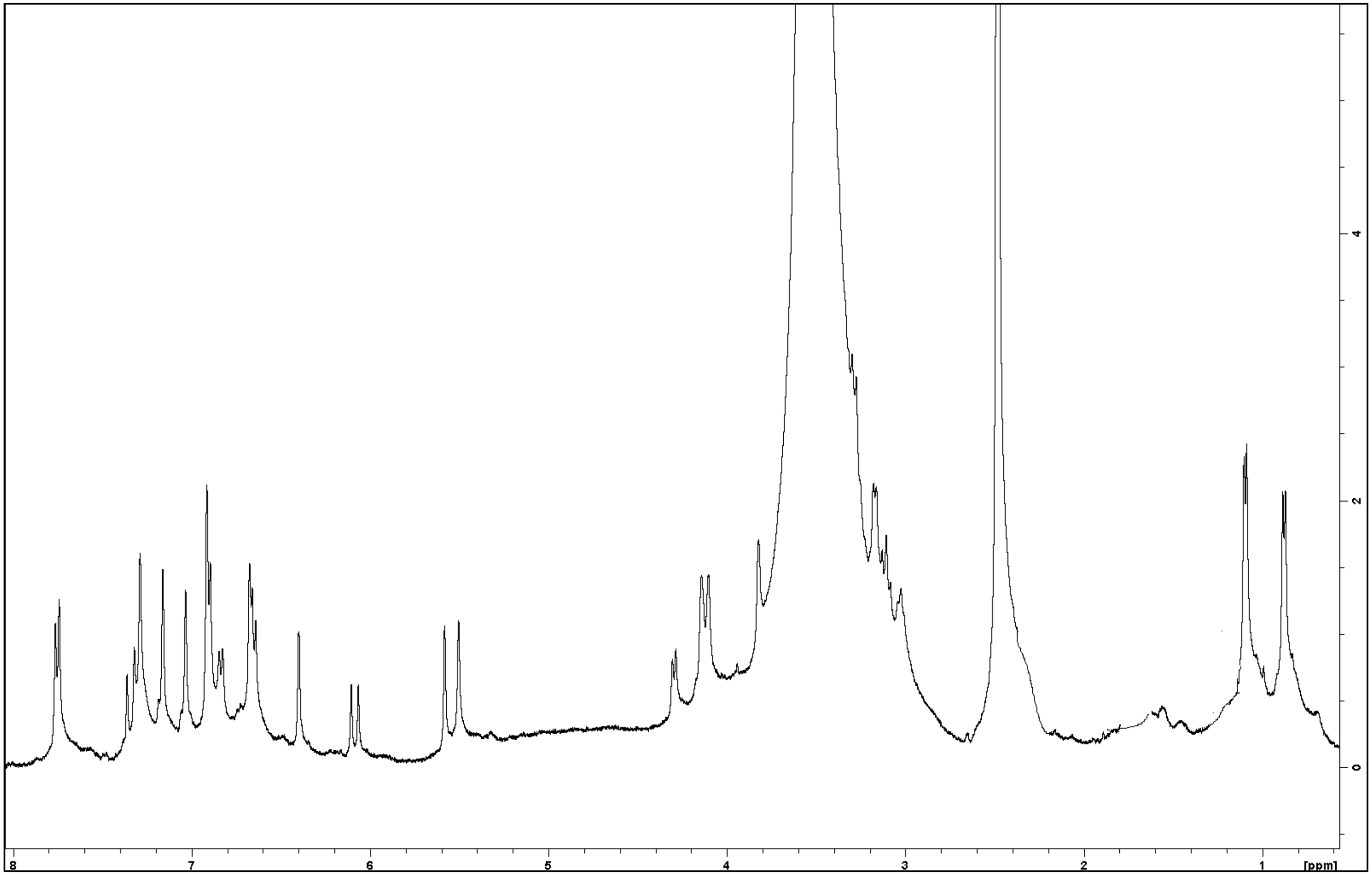

Figure S14. 100 MHz ^{13}C NMR spectrum of compound **6** in DMSO-d_6

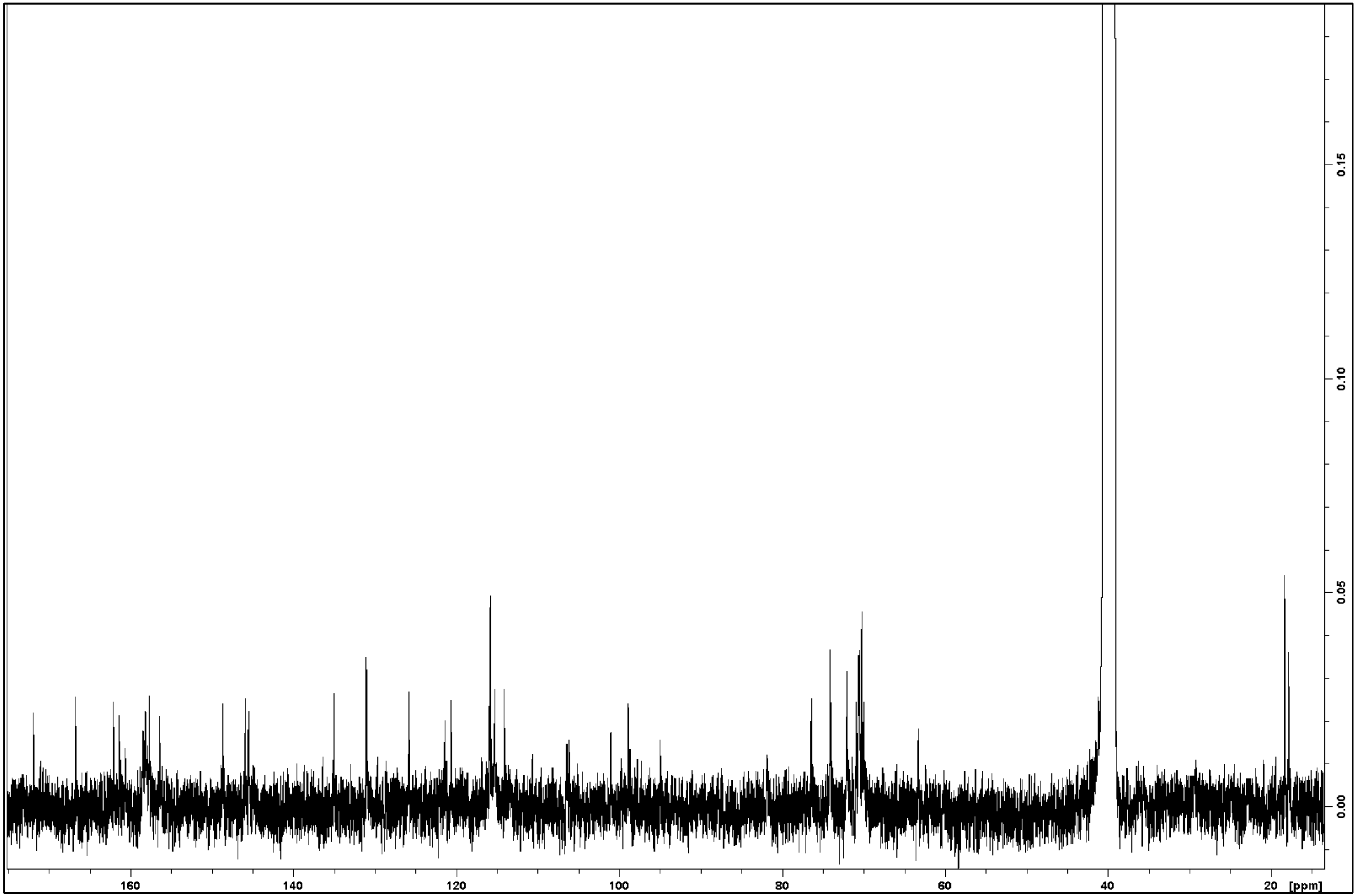

Figure S15. 400 MHz ^{1}H NMR spectrum of compound **7** in DMSO-d_6

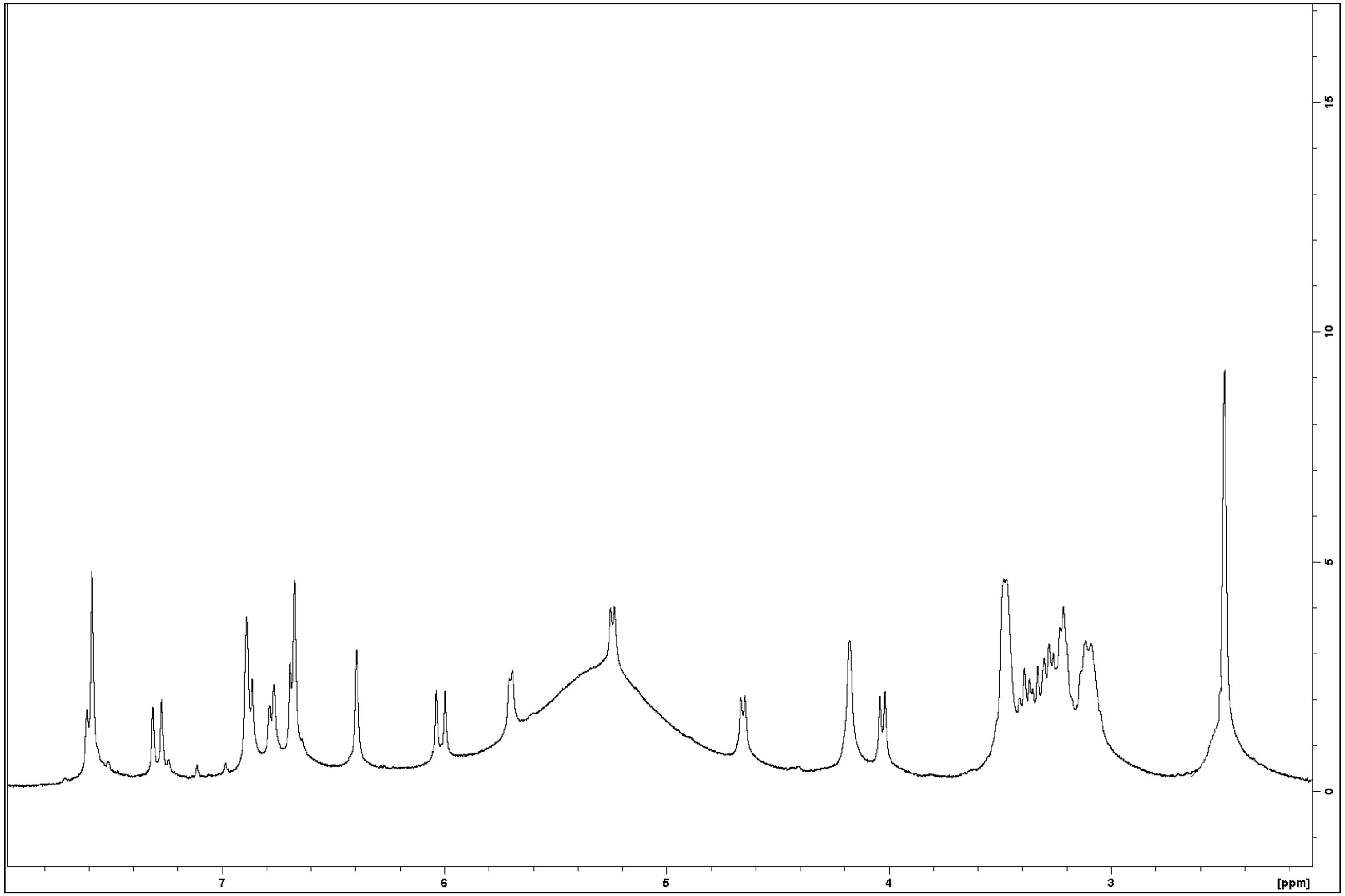

Figure S16. 100 MHz ^{13}C NMR spectrum of compound **7** in DMSO-d_6

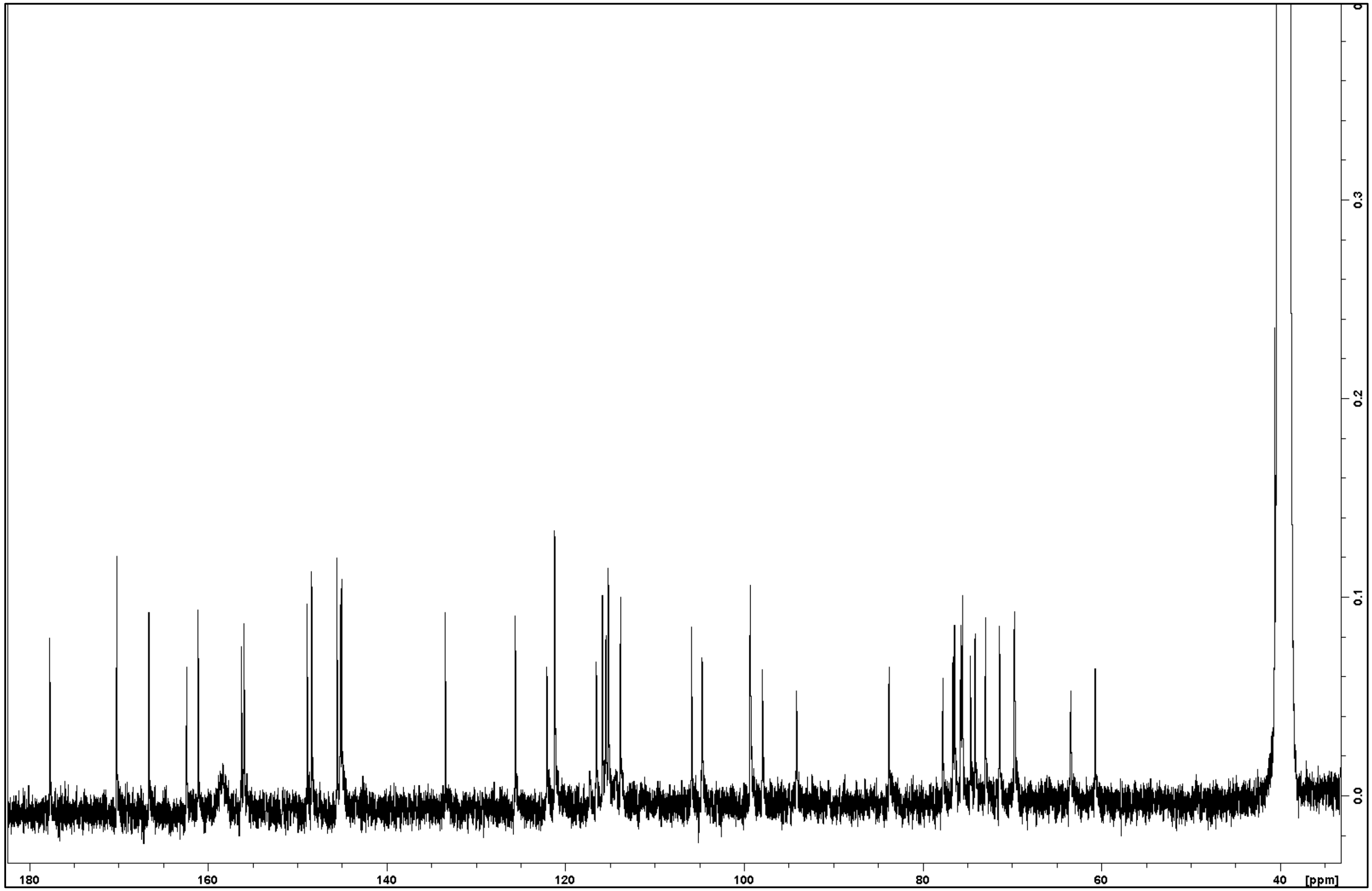

Figure S17. 400 MHz ^{1}H NMR spectrum of compound **8** in DMSO-d_6

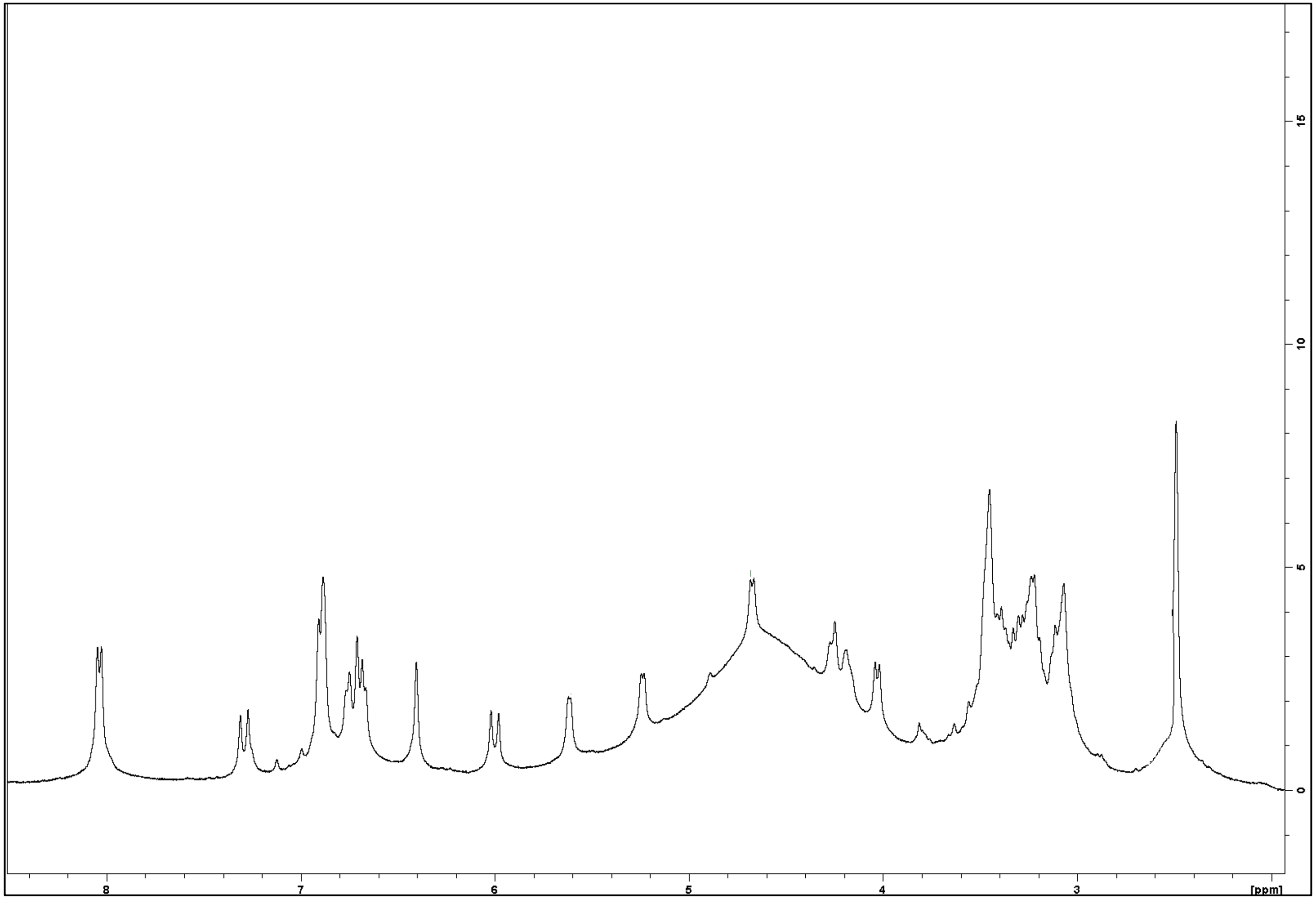

Figure S18. 100 MHz ^{13}C NMR spectrum of compound **8** in DMSO-d_6

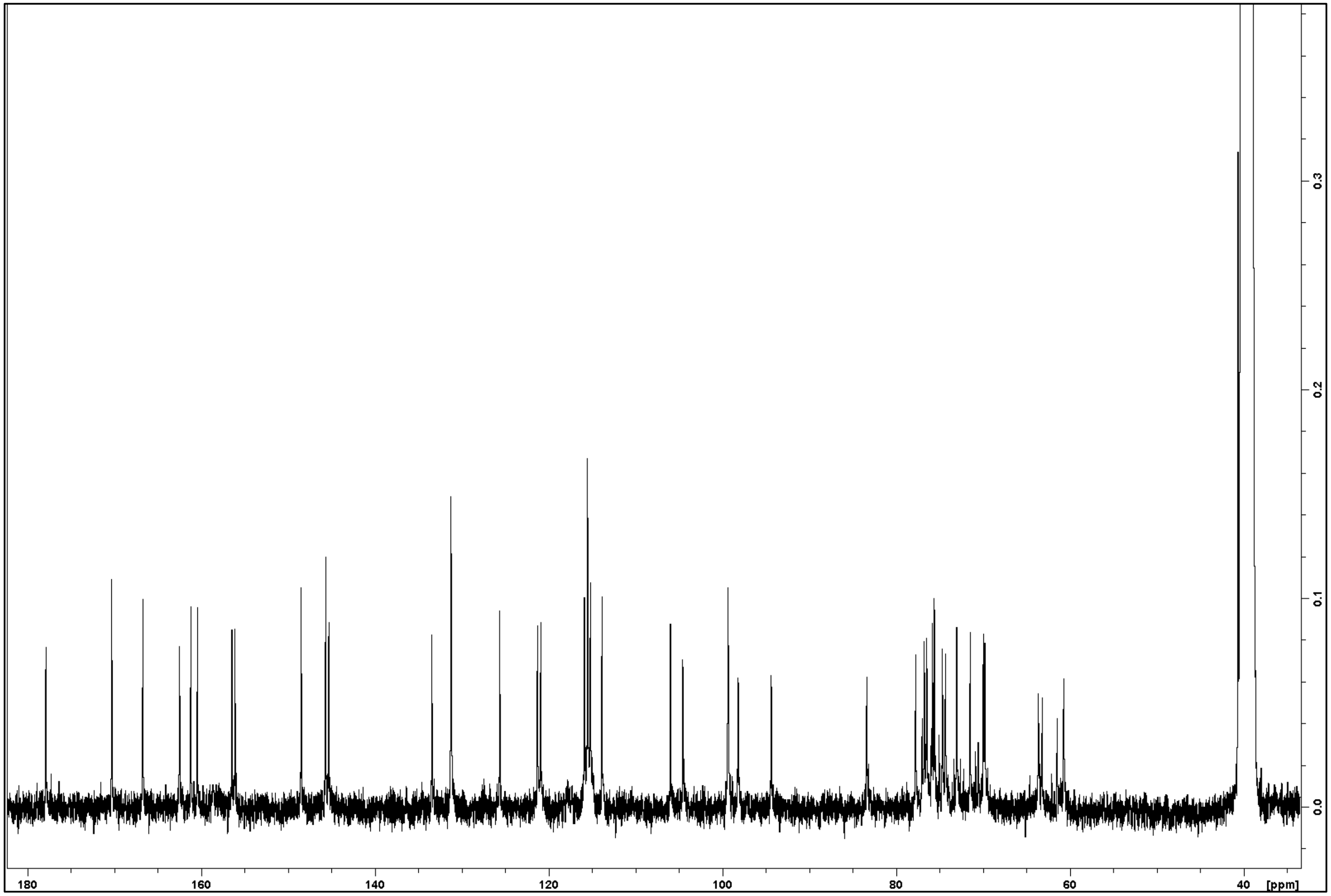

Figure S19. HPLC-PDA analysis of the reaction mixture of compound **4** with α-L-rhamosidase

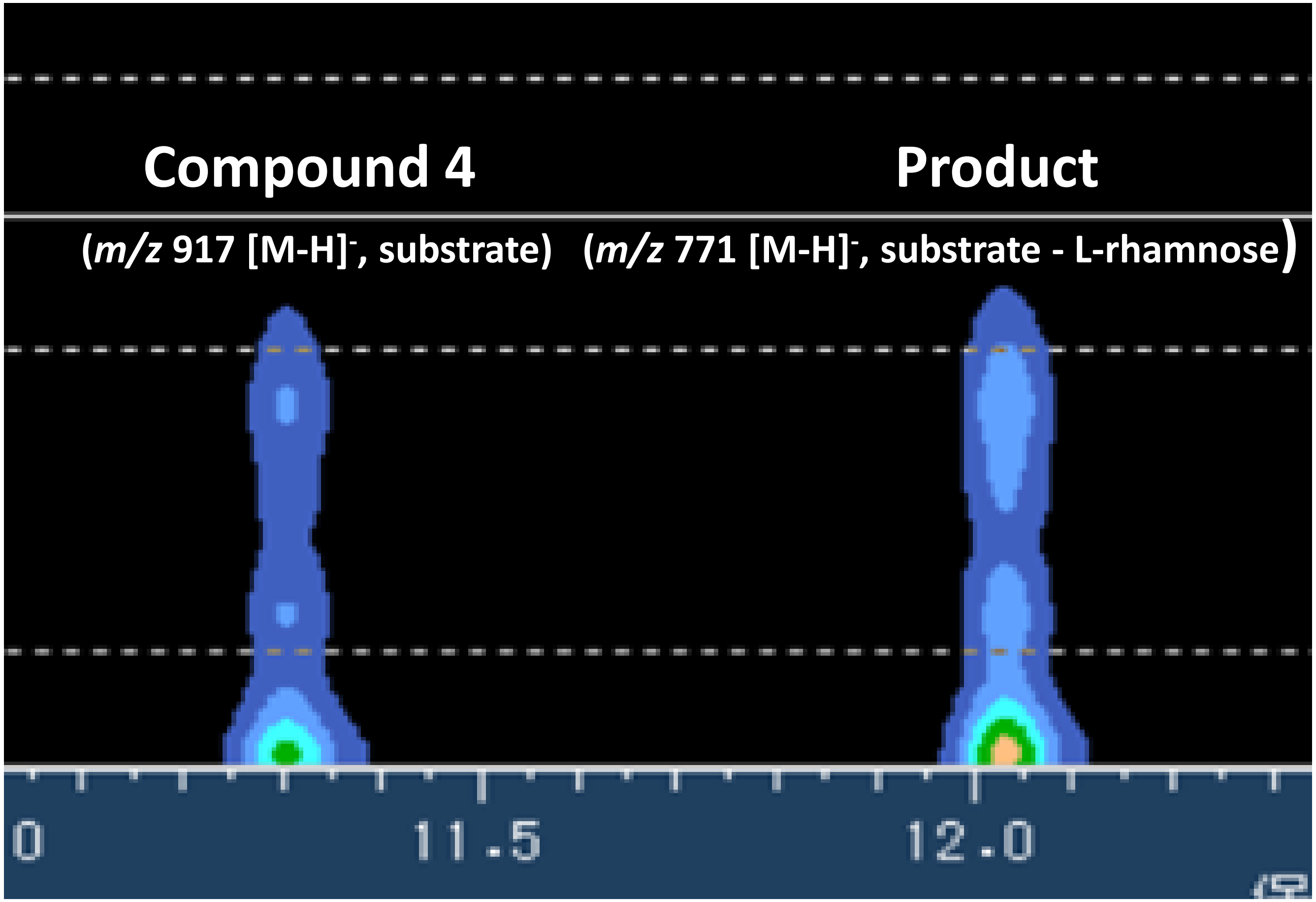

Figure S20. HPLC-PDA analysis of the reaction mixture of compound **8** with β-D-glucuronidase

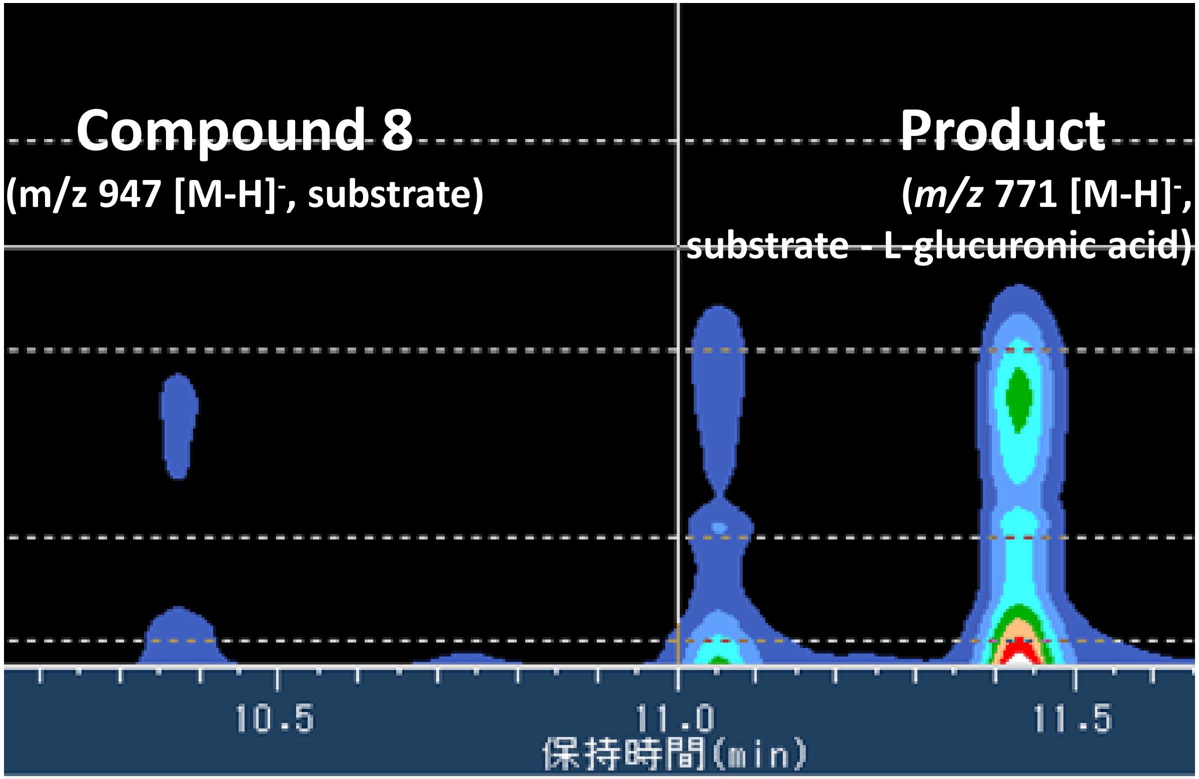

Figure S21. HPLC-PDA analysis of the extracts from the petals of yellow-flower cultivars, ‘f2’, ‘Kayak’, ‘Passat’, and ‘Boulevard’

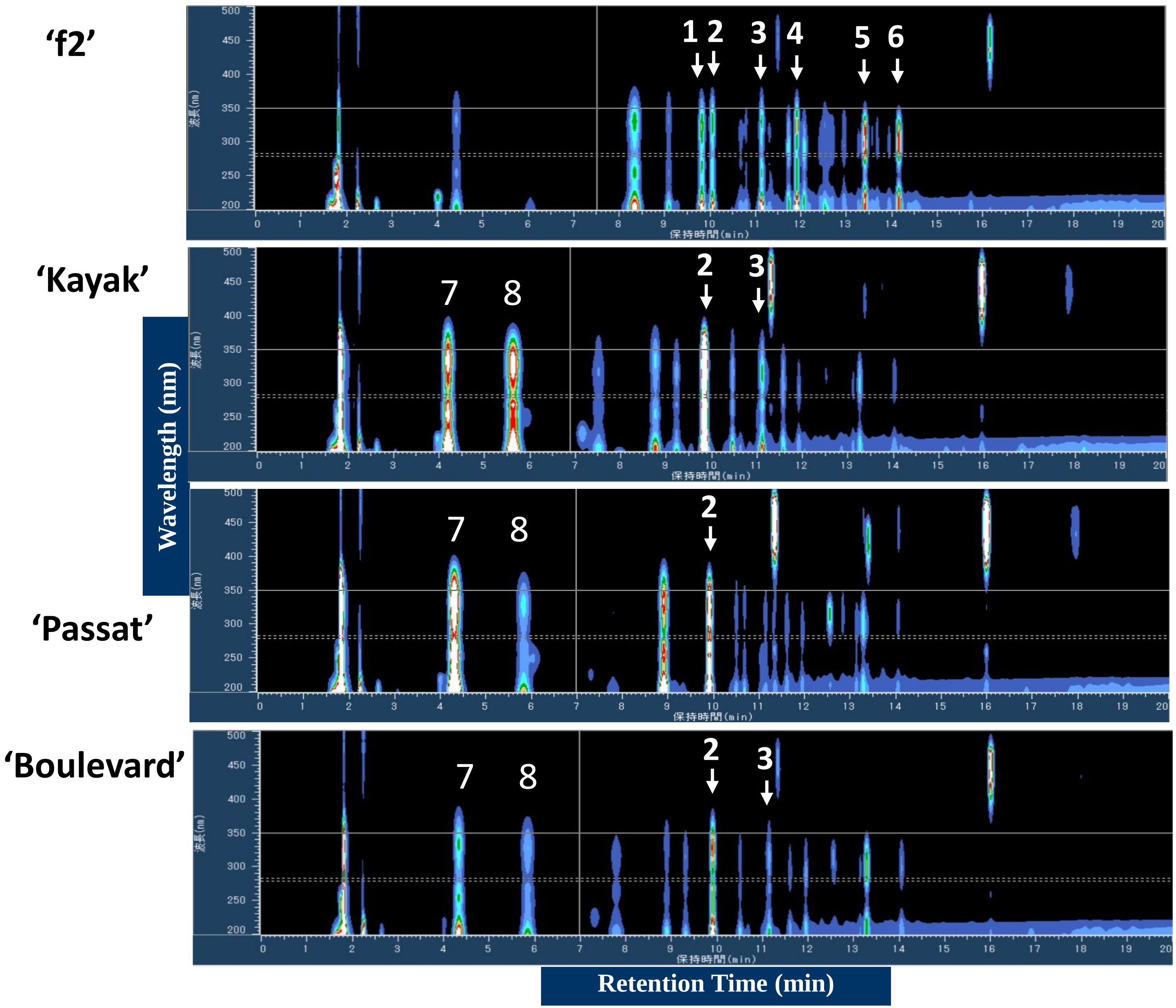

Figure S22. HPLC-PDA analysis of the extracts from the petals of cultivars ‘f2’, ‘Silk’ and ‘White’, and wild species ‘Alba’ and *F. refracta*

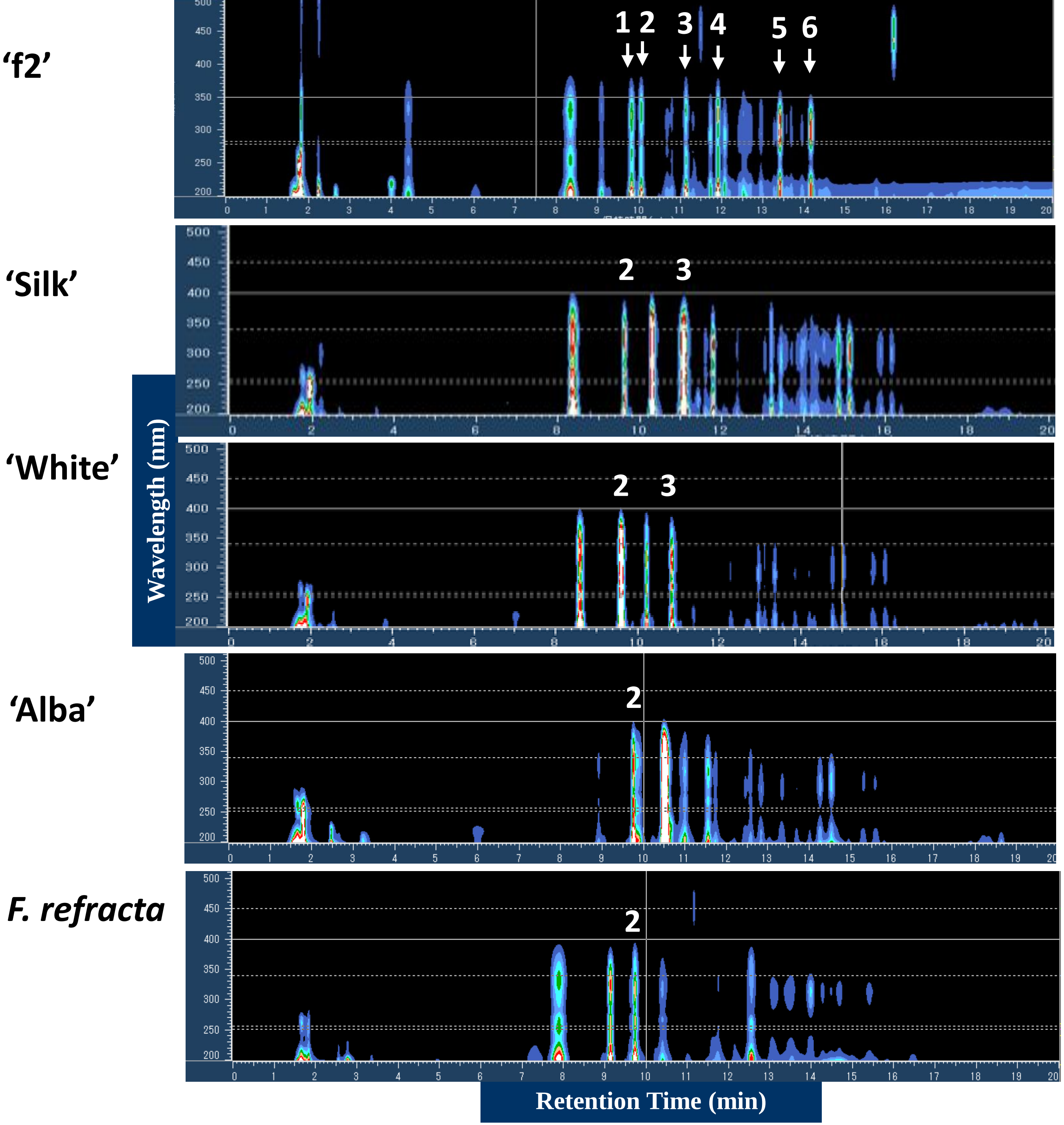

Physicochemical data S1.

Physicochemical data for compounds **1**

Yellow solid. $[a]_D^{24}$ -26.6° (*c* 0.1, MeOH). UV-vis (MeOH) λmax 254, 330 nm. HR-ESI-MS (positive) *m/z* 957.22643 $[M+Na]^+$ (calcd for $C_{42}H_{46}NaO_{24}$, 957.22767). ^1H NMR (DMSO-d_6) δ 0.91 (3H, d, *J* = 7.0 Hz, H-6’’’), 3.02 (1H, dd, *J* = 7.6, 8.5 Hz, H-2’’’’’), 3.11 (1H, m, H-4’’’), 3.15 (1H, m, H-3’’’’’), 3.21 (1H, m, H-5’’’’’), 3.27 (1H, m, H-3’’’’), 3.29 (1H, m, H-4’’’’’), 3.38 (1H, m, H-4’’’’), 3.39 (1H, m, H-6’’’’b), 3.56 (1H, m, H-5’’’), 3.57 (1H, m, H-3’’’), 3.64 (1H, m, H-6’’’’a), 3.99 (1H, m, H-6’’’’’b), 4.03 (1H, m, H-5’’’’), 4.04 (1H, dd, *J* = 6.7, 7.8 Hz, H-2’’’’), 4.13 (1H, d, J = 2.0 Hz, H-2’’’), 4.21 (1H, m, H-6’’’’’a), 4.26 (1H, d, J = 7.6 Hz, H-1’’’’’), 5.25 (1H, d, *J* = 6.7 Hz, H-1’’’’), 5.49 (1H, s, H-1’’’’), 6.17 (1H, d, *J* = 15.9 Hz, H-2’’), 6.42 (1H, s, H-6), 6.67 (1H, d, *J* = 8.1 Hz, H-8’’), 6.72 (1H, s, H-8), 6.89 (1H, d, *J* = 8.5 Hz, H-5’), 6.89 (1H, d, *J* = 8.1 Hz, H-9’’), 6.96 (1H, s, H-5’’), 7.27 (1H, d, *J* = 8.5 Hz, H-6’), 7.37 (1H, d, *J* = 15.9 Hz, H-3’’), 7.39 (s, H-2’). ^{13}C NMR (DMSO-d_6) δ 17.0 (C-6’’’), 61.3 (C-6’’’’), 62.8 (C-6’’’’’), 70.4 (C-5’’’), 70.6 (C-3’’’), 71.4 (C-4’’’’), 71.9 (C-3’’’’’), 73.0 (C-4’’’’’), 73.8 (C-2’’’’’), 74.0 (C-5’’’’’), 75.6 (C-3’’’’), 75.8 (C-5’’’’), 76.2 (C-2’’’’), 76.2 (C-4’’’), 81.7 (C-2’’’), 99.4 (C-1’’’’), 99.4 (C-6), 100.8 (C-1’’’), 106.0 (C-4a), 106.5 (C-1’’’’’), 114.1 (C-2’’), 115.1 (C-5’’), 115.8 (C-2’), 115.8 (C-5’), 115.8 (C-8’’), 120.6 (C-1’), 121.2 (C-9’’), 121.4 (C-6’), 125.7 (C-4’’), 134.7 (C-3), 145.3 (C-3’), 145.4 (C-3’’), 145.7 (C-6’’), 148.5 (C-7’’), 149.0 (C-4’), 156.2 (C-8a), 157.6 (C-2), 161.2 (C-5), 162.7 (C-7), 166.6 (C-1’’), 178.2 (C-4).

Physicochemical data S2.

Physicochemical data for compounds **2**

Yellow solid. [a]$_D^{24}$ -11.3° (*c* 0.1, MeOH). UV-vis (MeOH) λmax 254, 330 nm. HR-ESI-MS (positive) *m/z* 957.22591 $[M+Na]^+$ (calcd for $C_{42}H_{46}NaO_{24}$, 957.22767). ^{1}H NMR (DMSO-d_6) δ 1.11 (3H, d, *J* = 6.0 Hz, H-6’’’’), 3.09 (1H, m, H-5’’’), 3.11 (1H, dd, *J* = 7.7, 8.0 Hz, H-4’’’), 3.12 (1H, dd, *J* = 7.8, 8.9 Hz, H-2’’’’’), 3.15 (1H, dd, *J* = 9.4, 9.9 Hz, H-4’’’’’), 3.21 (1H, dd, *J* = 8.9, 9.4 Hz, H-3’’’’’), 3.24 (1H, m, H-6’’’b), 3.28 (1H, dd, *J* = 9.4, 9.8 Hz, H-4’’’’), 3.39 (1H, dq, *J* = 6.0, 9.8 Hz, H-5’’’’), 3.44 (1H, m, H-6’’’a), 3.46 (1H, dd, *J* = 5.4, 9.9 Hz, H-5’’’’’), 3.47 (2H, H-2’’’ and H-3’’’), 3.62 (1H, dd, *J* = 2.9, 9.4 Hz, H-3’’’’), 3.83 (1H, d, *J* = 2.9 Hz, H-2’’’’), 4.16 (1H, dd, *J* = 5.4, 11.6 Hz, H-6’’’’’b), 4.20 (1H, d, *J* = 11.6 Hz, H-6’’’’’a), 4.63 (1H, d, *J* = 7.8 Hz, H-1’’’’’), 5.49 (1H, s, H-1’’’’), 5.71 (1H, d, *J* = 6.5 Hz, H-1’’’), 5.96 (1H, d, *J* = 15.9 Hz, H-2’’), 6.40 (1H, s, H-6), 6.60 (1H, s, H-8), 6.65 (1H, d, *J* = 8.3 Hz, H-8’’), 6.72 (1H, d, *J* = 8.3 Hz, H-9’’), 6.85 (1H, s, H-5’’), 6.86 (1H, d, *J* = 8.5 Hz, H-5’), 7.58 (1H, s, H-2’), 7.25 (1H, d, *J* = 15.9 Hz, H-3’’), 7.61 (1H, d, *J* = 8.5 Hz, H-6’). ^{13}C NMR (DMSO-d_6) δ 18.1 (C-6’’’’), 60.7 (C-6’’’), 63.4 (C-6’’’’’), 69.7 (C-4’’’), 69.9 (C-2’’’’), 70.0 (C-4’’’’’), 70.2 (C-5’’’’), 70.4 (C-3’’’’), 71.8 (C-4’’’’), 74.1 (C-5’’’’’), 74.7 (C-2’’’’’), 76.4 (C-3’’’’’), 76.6 (C-3’’’), 77.8 (C-5’’’), 83.9 (C-2’’’), 94.0 (C-8), 98.0 (C-1’’’), 98.5 (C-1’’’’), 99.5 (C-6), 104.8 (C-1’’’’’), 105.7 (C-4a), 113.7 (C-2’’), 115.1 (C-5’’), 115.5 (C-5’), 115.5 (C-8’’), 116.3 (C-2’), 121.1 (C-9’’), 121.2 (C-1’), 122.1 (C-6’), 125.5 (C-4’’), 133.4 (C-3), 145.1 (C-3’), 145.1 (C-3’’), 145.5 (C-6’’), 148.4 (C-7’’), 148.8 (C-4’), 155.9 (C-8a), 156.0 (C-2), 161.0 (C-5), 161.6 (C-7), 166.5 (C-1’’), 177.6 (C-4).

Physicochemical data S3.

Physicochemical data for compounds **3**

Yellow solid. $[a]_D^{24}$ -29.4° (*c* 0.1, MeOH). UV-vis (MeOH) λmax 245, 268, 327 nm. HR-ESI-MS (positive) *m/z* 941.23371 $[M+Na]^+$ (calcd for $C_{42}H_{46}NaO_{23}$, 941.23278). ^1H NMR (DMSO-d_6) δ 1.10 (3H, d, *J* = 6.1 Hz, H-6''''), 3.06 (1H, dd, *J* = 7.7, 8.0 Hz, H-4'''), 3.07 (1H, m, H-5'''), 3.11 (1H, dd, *J* = 7.6, 8.9 Hz, H-2'''''), 3.15 (1H, dd, *J* = 9.4, 9.9 Hz, H-4'''''), 3.22 (1H, dd, *J* = 8.9, 9.4 Hz, H-3'''''), 3.24 (1H, m, H-6'''b), 3.28 (1H, dd, *J* = 9.4, 9.8 Hz, H-4''''), 3.39 (1H, dq, *J* = 6.1, 9.8 Hz, H-5''''), 3.44 (1H, dd, *J* = 7.0, 8.3 Hz, H-2'''), 3.45 (1H, dd, *J* = 5.4, 9.9 Hz, H-5'''''), 3.47 (1H, dd, *J* = 7.7, 8.3 Hz, H-3'''), 3.47 (1H, m, H-6'''a), 3.62 (1H, dd, *J* = 2.0, 9.4 Hz, H-3''''), 3.83 (1H, d, *J* = 2.0 Hz, H-2''''), 4.16 (1H, dd, *J* = 5.4, 11.6 Hz, H-6'''''b), 4.26 (1H, d, *J* = 11.6 Hz, H-6'''''a), 4.64 (1H, d, *J* = 7.6 Hz, H-1'''''), 5.49 (1H, s, H-1''''), 5.60 (1H, d, *J* = 7.0 Hz, H-1'''), 5.96 (1H, d, *J* = 15.9 Hz, H-2''), 6.35 (1H, d, *J* = 2.0 Hz, H-6), 6.63 (1H, d, *J* = 8.7 Hz, H-8''), 6.64 (1H, d, *J* = 2.0 Hz, H-8), 6.71 (1H, d, *J* = 8.7 Hz, H-9''), 6.83 (1H, s, H-5''), 6.88 (2H, d, *J* = 8.8 Hz, H-3' and H-5'), 7.25 (1H, d, *J* = 15.9 Hz, H-3''), 8.04 (2H, d, *J* = 8.8 Hz, H-2' and H-6'). ^{13}C NMR (DMSO-d_6) δ 18.1 (C-6''''), 60.6 (C-6'''), 63.6 (C-6'''''), 69.7 (C-4'''), 70.0 (C-2''''), 70.0 (C-4'''''), 70.2 (C-4''''), 70.4 (C-3''''), 71.8 (C-5''''), 74.2 (C-5'''''), 74.7 (C-2'''''), 76.4 (C-3'''''), 76.6 (C-3'''), 77.7 (C-5'''), 83.5 (C-2'''), 94.3 (C-8), 98.2 (C-1'''), 98.5 (C-1''''), 99.5 (C-6), 104.6 (C-1'''''), 105.7 (C-4a), 113.7 (C-2''), 115.0 (C-5''), 115.4 (C-3' and C-5'), 115.5 (C-8''), 120.8 (C-1'), 121.1 (C-9''), 125.5 (C-4''), 131.2 (C-2' and C-6'), 133.4 (C-3), 145.1 (C-3''), 145.5 (C-6''), 148.4 (C-7''), 156.0 (C-4'), 156.1 (C-8a), 160.3 (C-2), 161.0 (C-5), 161.6 (C-7), 166.5 (C-1''), 177.7 (C-4).

Physicochemical data S4.

Physicochemical data for compounds **4**

Yellow solid. $[a]_D^{24}$ -22.9° (*c* 0.1, MeOH). UV-vis (MeOH) λmax 254, 330 nm. HR-ESI-MS (positive) *m/z* 941.23392 $[M+Na]^+$ (calcd for $C_{42}H_{46}NaO_{23}$, 941.23278). ^{1}H NMR (DMSO-d_6) δ 0.91 (3H, d, *J* = 6.0 Hz, H-6’’’), 1.10 (3H, d, *J* = 6.0 Hz, H-6’’’’), 3.02 (1H, dd, *J* = 7.5, 7.6 Hz, H-2’’’’’), 3.12 (1H, m, H-4’’’), 3.16 (1H, m, H-3’’’’’), 3.22 (1H, m, H-5’’’’’), 3.28 (1H, dd, *J* = 9.4, 9.4 Hz, H-4’’’’), 3.30 (1H, m, H-4’’’’’), 3.40 (1H, dq, *J* = 6.0, 9.4 Hz, H-5’’’’), 3.58 (1H, dd, *J* = 2.0, 9.4 Hz, H-3’’’), 3.60 (1H, dd, *J* = 3.0, 9.4 Hz, H-3’’’’), 3.60 (1H, m, H-5’’’), 3.83 (1H, d, *J* = 3.0 Hz, H-2’’’’), 3.99 (1H, d, *J* = 11.8 Hz, H-6’’’’’b), 4.15 (1H, dd, *J* = 2.0, 11.8 Hz, H-6’’’’’a), 4.15 (1H, d, *J* = 2.0 Hz, H-2’’’), 4.26 (1H, d, *J* = 7.5 Hz, H-1’’’’’), 5.48 (1H, s, H-1’’’), 5.51 (1H, s, H-1’’’’), 6.14 (1H, d, *J* = 15.9 Hz, H-2’’), 6.39 (1H, s, H-6), 6.66 (1H, d, *J* = 8.2 Hz, H-8’’), 6.68 (1H, s, H-8), 6.88 (1H, d, *J* = 8.0 Hz, H-5’), 6.88 (1H, d, *J* = 8.2 Hz, H-9’’), 6.94 (1H, s, H-5’’), 7.29 (1H, d, *J* = 8.0 Hz, H-6’), 7.36 (1H, d, *J* = 15.9 Hz, H-3’’), 7.40 (1H, s, H-2’). ^{13}C NMR (DMSO-d_6) δ 17.6 (C-6’’’), 18.1 (C-6’’’’), 62.8 (C-6’’’’’), 69.4 (C-2’’’’), 70.0 (C-3’’’’), 70.2 (C-5’’’’), 70.4 (C-5’’’), 70.6 (C-3’’’), 71.8 (C-4’’’’), 71.9 (C-4’’’), 71.9 (C-3’’’’’), 73.8 (C-2’’’’’), 73.9 (C-5’’’’’), 76.1 (C-4’’’’’), 81.8 (C-2’’’), 94.6 (C-8), 98.6 (C-1’’’’), 99.5 (C-6), 100.9 (C-1’’’), 105.8 (C-4a), 106.4 (C-1’’’’’), 114.0 (C-2’’), 114.5 (C-5’’), 115.1 (C-5’), 115.8 (C-2’), 115.8 (C-8’’), 120.6 (C-9’’), 121.1 (C-1’), 121.2 (C-6’), 125.6 (C-4’’), 134.8 (C-3), 145.3 (C-3’’), 145.4 (C-3’), 145.7 (C-6’’), 148.4 (C-7’’), 149.0 (C-4’), 156.1 (C-8a), 157.3 (C-2), 161.1 (C-5), 161.8 (C-7), 166.5 (C-1’’), 178.2 (C-4).

Physicochemical data S5.

Physicochemical data for compounds **5**

Yellow solid. [a]$_D^{24}$ -14.6° (*c* 0.1, MeOH). UV-vis (MeOH) λmax 254, 330 nm. HR-ESI-MS (positive) *m/z* 811.17077 [M+Na]$^+$ (calcd for $C_{36}H_{36}NaO_{20}$, 811.16976). ^{1}H NMR (DMSO-d_6) δ 3.08 (1H, m, H-5’’’), 3.10 (1H, m, H-4’’’’), 3.12 (1H, m, H-2’’’’), 3.20 (1H, m, H-4’’’), 3.21 (1H, m, H-3’’’’), 3.25 (1H, m, H-6’’’b), 3.46 (1H, m, H-5’’’’), 3.47 (1H, m, H-2’’’), 3.48 (1H, m, H-3’’’), 3.49 (1H, m, H-6’’’a), 4.13 (1H, m, H-6’’’’b), 4.20 (1H, m, H-6’’’’a), 4.66 (1H, d, *J* = 7.5 Hz, H-1’’’’), 5.70 (1H, d, *J* = 6.8 Hz, H-1’’’), 5.99 (1H, d, *J* = 15.9 Hz, H-2’’), 6.15 (1H, d, *J* = 1.5 Hz, H-6), 6.31 (1H, d, *J* = 1.5 Hz, H-8), 6.68 (1H, d, *J* = 8.2 Hz, H-8’’), 6.76 (1H, dd, *J* = 1.0, 8.2 Hz, H-8’’), 6.85 (1H, d, *J* = 8.4 Hz, H-5’), 6.89 (1H, d, *J* = 1.0 Hz, H-5’’), 7.29 (1H, d, *J* = 15.9 Hz, H-3’’), 7.51 (1H, d, *J* = 1.9 Hz, H-2’), 7.58 (1H, dd, *J* = 1.9, 8.4 Hz, H-6’). ^{13}C NMR (DMSO-d_6) δ 60.7 (C-6’’’), 64.0 (C-6’’’’), 69.7 (C-4’’’), 69.8 (C-4’’’’), 74.1 (C-5’’’’), 74.6 (C-2’’’’), 76.4 (C-3’’’’), 76.6 (C-3’’’), 77.7 (C-5’’’), 83.7 (C-2’’’), 93.6 (C-8), 98.0 (C-1’’’), 98.7 (C-6), 104.0 (C-4a), 104.7 (C-1’’’’), 113.8 (C-2’’), 115.3 (C-5’’), 115.5 (C-5’), 115.8 (C-8’’), 116.3 (C-2’), 120.9 a145.6 (C-6’’), 148.4 (C-7’’), 148.7 (C-4’), 155.5 (C-2), 156.3 (C-8a), 161.4 (C-5), 164.1 (C-7), 166.6 (C-1’’), 177.5 (C-4).

Physicochemical data S6.

Physicochemical data for compounds **6**
Yellow solid. $[a]_D^{24}$ -32.7° (*c* 0.1, MeOH). UV-vis (MeOH) λmax 245, 268, 327 nm. HR-ESI-MS (positive) *m/z* 925.23411 $[M+Na]^+$ (calcd for $C_{42}H_{46}NaO_{22}$, 925.23787). ^1H NMR (DMSO-d_6) δ 0.88 (3H, d, *J* = 5.8 Hz, H-6’’’), 1.10 (3H, d, *J* = 5.9 Hz, H-6’’’’), 3.02 (1H, m, H-2’’’’’), 3.16 (1H, m, H-3’’’’’), 3.30 (1H, m, H-2’’’’), 3.22 (1H, m, H-5’’’’’), 3.28 (1H, m, H-4’’’’), 3.30 (1H, m, H-4’’’’’), 3.33 (1H, m, H-5’’’), 3.39 (1H, m, H-5’’’’), 3.59 (1H, m, H-3’’’), 3.60 (1H, m, H-4’’’), 3.62 (1H, m, H-3’’’’), 4.00 (1H, m, H-6’’’’’b), 4.15 (1H, m, H-6’’’’’a), 4.15 (1H, d, *J* = 2.0 Hz, H-2’’’), 4.30 (1H, d, *J* = 7.4 Hz, H-1’’’’’), 5.51 (1H, s, H-1’’’’), 5.59 (1H, s, H-1’’’), 6.09 (1H, d, *J* = 15.4 Hz, H-2’’), 6.41 (1H, s, H-6), 6.66 (1H, d, *J* = 7.3 Hz, H-9’’), 6.68 (1H, s, H-8), 6.84 (1H, d, *J* = 7.3 Hz, H-8’’), 6.91 (2H, d, *J* = 8.3 Hz, H-3’ and H-5’), 6.92 (1H, s, H-5’’), 7.35 (1H, d, *J* = 15.8 Hz, H-3’’), 7.76 (2H, d, *J* = 8.3 Hz, H-2’ and H-6’). ^{13}C NMR (DMSO-d_6) δ 17.6 (C-6’’’), 18.1 (C-6’’’’), 63.0 (C-6’’’’’), 70.0 (C-2’’’’), 70.3 (C-3’’’’), 70.3 (C-5’’’’), 70.4 (C-4’’’), 70.6 (C-3’’’), 71.4 (C-5’’’), 71.8 (C-3’’’’’), 71.8 (C-4’’’’), 73.9 (C-2’’’’’), 73.9 (C-5’’’’’), 76.2 (C-4’’’’’), 81.8 (C-2’’’), 94.8 (C-8), 98.6 (C-1’’’’), 99.6 (C-6), 100.8 (C-1’’’), 105.9 (C-4a), 105.9 (C-1’’’’’), 113.9 (C-2’’), 115.1 (C-5’’), 115.1 (C-8’’), 115.6 (C-3’ and C-5’), 120.4 (C-9’’), 121.2 (C-1’), 125.6 (C-4’’), 130.8 (C-2’ and C-6’), 134.8 (C-3), 145.3 (C-6’’), 145.6 (C-3’’), 148.4 (C-7’’), 156.2 (C-8a), 157.4 (C-4’), 160.5 (C-2), 161.1 (C-5), 161.9 (C-7), 166.5 (C-1’’), 178.1 (C-4).

Physicochemical data S7.

Physicochemical data for compounds **7**

Yellow solid. $[a]_D^{24}$ -63.5° (c 0.1, MeOH). UV-vis (MeOH) λmax 254, 330 nm. HR-ESI-MS (negative) *m/z* 963.20260 $[M-H]^-$ (calcd for $C_{42}H_{43}O_{26}$, 963.20427). ^1H NMR (DMSO-d_6) δ 3.09 (1H, m, H-5’’’), 3.11 (1H, m, H-4’’’), 3.12 (1H, m, H-2’’’’’), 3.21 (1H, m, H-3’’’’’), 3.22 (1H, m, H-4’’’’’), 3.24 (1H, m, H-6’’’b), 3.29 (1H, m, H-2’’’’), 3.34 (1H, m, H-3’’’’), 3.39 (1H, m, H-4’’’’), 3.46 (1H, m, H-5’’’’’), 3.47 (1H, m, H-3’’’), 3.48 (1H, m, H-6’’’a), 3.48 (1H, dd, *J* = 6.5, 8.3 Hz, H-2’’’), 4.03 (1H, d, *J* = 9.2 Hz, H-5’’’’), 4.18 (2H, H-6’’’’’b and H-6’’’’’a), 4.66 (1H, d, *J* = 7.4 Hz, H-1’’’’’), 5.24 (1H, d, *J* = 7.9 Hz, H-1’’’’), 5.69 (1H, d, *J* = 6.5 Hz, H-1’’’), 6.02 (1H, d, *J* = 15.8 Hz, H-2’’), 6.39 (1H, s, H-6), 6.67 (1H, s, H-8), 6.69 (1H, d, *J* = 7.9 Hz, H-8’’), 6.78 (1H, d, *J* = 7.4 Hz, H-9’’), 6.88 (1H, d, *J* = 8.7 Hz, H-5’), 6.89 (1H, s, H-5’’), 7.29 (1H, d, *J* = 15.8 Hz, H-3’’), 7.59 (1H, s, H-2’), 7.60 (1H, d, *J* = 8.7 Hz, H-6’). ^{13}C NMR (DMSO-d_6) δ 60.7 (C-6’’’), 63.4 (C-6’’’’’), 69.4 (C-4’’’’’), 69.7 (C-4’’’), 71.4 (C-4’’’’), 73.0 (C-2’’’’), 74.1 (C-5’’’’’), 74.7 (C-2’’’’’), 75.6 (C-3’’’’), 75.7 (C-5’’’’), 76.4 (C-3’’’’’), 76.6 (C-3’’’), 77.7 (C-5’’’), 83.8 (C-2’’’), 94.1 (C-8), 97.9 (C-1’’’), 99.3 (C-1’’’’), 99.3 (C-6), 104.7 (C-1’’’’’), 105.9 (C-4a), 113.8 (C-2’’), 115.2 (C-5’’), 115.5 (C-5’), 115.9 (C-8’’), 116.5 (C-2’), 121.2 (C-9’’), 121.2 (C-1’), 122.1 (C-6’), 125.6 (C-4’’), 133.4 (C-3), 145.0 (C-3’), 145.2 (C-3’’), 145.6 (C-6’’), 148.4 (C-7’’), 148.9 (C-4’), 156.0 (C-8a), 156.3 (C-2), 161.1 (C-5), 162.4 (C-7), 166.6 (C-1’’), 170.2 (C-6’’’’), 177.7 (C-4).

Physicochemical data S8.

Physicochemical data for compounds **8**

Yellow solid. $[a]_D24$ -52.1° (*c* 0.1, MeOH). UV-vis (MeOH) λmax 245, 268, 327 nm. HR-ESI-MS (negative) *m/z* 947.20790 $[M-H]^-$ (calcd for $C_{42}H_{43}O_{26}$, 947.20935). ^1H NMR (DMSO-d_6) δ 3.07 (1H, m, H-4’’’’’), 3.08 (1H, m, H-5’’’), 3.12 (1H, m, H-2’’’’’), 3.21 (1H, m, H-4’’’), 3.24 (1H, m, H-6’’’b), 3.29 (1H, m, H-2’’’’), 3.34 (1H, m, H-3’’’’), 3.39 (1H, m, H-4’’’’), 3.46 (4H, H-2’’’, H-3’’’, H-3’’’’’, and H-5’’’’’), 3.47 (1H, m, H-6’’’a), 4.03 (1H, m, H-5’’’’), 4.17 (1H, m, H-6’’’’’b), 4.28 (1H, m, H-6’’’’’a), 4.67 (1H, d, *J* = 7.1 Hz, H-1’’’’’), 5.24 (1H, d, *J* = 5.2 Hz, H-1’’’’), 5.62 (1H, m, H-1’’’), 6.00 (1H, d, *J* = 15.6 Hz, H-2’’), 6.40 (1H, s, H-6), 6.74 (1H, s, H-8), 6.76 (1H, d, *J* = 7.9 Hz, H-9’’), 6.89 (1H, s, H-5’’), 6.89 (1H, d, *J* = 7.9 Hz, H-8’’), 6.90 (2H, d, *J* = 7.8 Hz, C-3’ and C-5’), 7.29 (1H, d, *J* = 15.6 Hz, H-3’’), 8.03 (2H, d, *J* = 7.8 Hz, C-2’ and C-6’). ^{13}C NMR (DMSO-d_6) δ 60.7 (C-6’’’), 63.6 (C-6’’’’’), 69.7 (C-4’’’’’), 69.9 (C-4’’’), 71.4 (C-4’’’’), 73.0 (C-2’’’’), 74.3 (C-5’’’’’), 74.6 (C-2’’’’’), 75.6 (C-5’’’’), 75.8 (C-3’’’’), 76.4 (C-3’’’’’), 76.7 (C-3’’’), 77.7 (C-5’’’), 83.3 (C-2’’’), 94.3 (C-8), 98.1 (C-1’’’), 99.3 (C-1’’’’), 99.3 (C-6), 104.5 (C-1’’’’’), 105.9 (C-4a), 113.8 (C-2’’), 115.1 (C-5’’), 115.5 (C-3’ and C-5’), 115.8 (C-8’’), 120.9 (C-1’), 121.2 (C-9’’), 125.6 (C-4’’), 131.2 (C-2’ and C-5’), 133.4 (C-3), 145.2 (C-6’’), 145.6 (C-3’’), 148.4 (C-7’’), 156.1 (C-8a), 156.4 (C-4’), 160.4 (C-2), 161.1 (C-5), 162.4 (C-7), 166.6 (C-1’’), 170.2 (C-6’’’’), 177.8 (C-4).
